# Gold- and Platinum-Peptide Bioconjugates in Cancer Therapy: Recent Advances and Future Directions

**DOI:** 10.3390/pharmaceutics18070794

**Published:** 2026-06-28

**Authors:** Anna Giorgio, Vincenzo Abagnale, Michele Saviano, Annarita Del Gatto, Laura Zaccaro

**Affiliations:** 1 Institute of Biostructure and Bioimaging (IBB), National Research Council (CNR), 80131 Naples, Italy; annagiorgio@cnr.it (A.G.); vincenzo.abagnale@unicampania.it (V.A.); 2 Interuniversity Research Centre on Bioactive Peptides (CIRPeB) “Carlo Pedone”, University of Naples, 80131 Naples, Italy; 3 Department of Environmental, Biological and Pharmaceutical Sciences and Technologies, University of Campania “L. Vanvitelli”, 81100 Caserta, Italy; 4 Institute of Crystallography (IC), CNR, 81100 Caserta, Italy; michele.saviano@cnr.it

**Keywords:** metal-peptide bioconjugate, platinum complex, gold complex, drug delivery, cancer therapy

## Abstract

**Background**: Metal-based anticancer drugs, particularly platinum and gold complexes, play a central role in chemotherapy but are often limited by systemic toxicity, resistance, and suboptimal selectivity. Peptide conjugation has emerged as a versatile strategy to modulate the pharmacokinetic and biological properties of metal complexes, enabling targeted delivery, improved uptake, and controlled activation. This review aims to critically analyze platinum- and gold-peptide bioconjugates in cancer therapy, focusing on directly reactive metal complexes and redox-activated prodrug systems. **Methods**: Relevant literature from the past two decades was surveyed across major scientific databases, focusing on the design, conjugation strategies, biological activity, and mechanisms of action of Pt- and Au-peptide bioconjugates. **Results**: Reviewed studies reveal distinct behavior for platinum- and gold-based systems. Pt(II)-peptide conjugates primarily retain DNA-reactive interaction, with peptides mainly enhancing cellular uptake, selective targeting and solubility, although improved cytotoxicity is not consistently achieved. In contrast, Pt(IV)-peptide conjugates function as prodrugs, where axial peptide functionalization allows greater structural versatility and sometimes improved selectivity, with therapeutic efficacy strongly depending on intracellular reduction kinetics. Au(I)-peptide conjugates act as directly reactive species targeting thiol- and selenol-containing proteins, whereas Au(III) bioconjugates often behave as redox-activated prodrugs, with peptide conjugation influencing stability and cellular fate. **Conclusions**: Overall, peptide conjugation represents a powerful but non-trivial approach for optimizing metal-based anticancer agents. The success of metal-peptide bioconjugates critically depends on balancing peptide-mediated delivery with the intrinsic reactivity and activation pathways of the metal center. A function-guided design of bioconjugates is essential to achieve genuine selectivity and therapeutic benefit.

## 1. Introduction

Given the prevalence of metals within the periodic table, it is unsurprising that they participate extensively in fundamental biological processes, whether as free ions, salts and/or constituents of coordination complexes [1]. Several metals are indispensable for sustaining life, whereas others exhibit therapeutically and diagnostically relevant properties. Deviations from the tightly regulated concentrations of essential metals can give rise to physiological disorders, acute toxicity, or increased susceptibility to infectious diseases [2]. Compounds incorporating non-essential metal ions may, in some cases, be tolerated by organisms at unexpectedly high levels, thereby rendering them useful in biomedical applications, such as chemotherapeutic agents (platinum-based chemotherapeutics) [3], diagnostic tracers (gadolinium-based MRI contrast agents) [4] and metal-based formulations [5,6,7]. Conversely, certain metal-based species display pronounced antimicrobial or antiviral activity even at minimal concentrations, further underscoring their potential clinical relevance. In addition, numerous metals are integral components of radiopharmaceuticals (RPs) useful in nuclear medicine, as radiotherapeutic or contrast agents.

### 1.1. Platinum and Gold Complexes in Biomedicine

**Figure 1:**
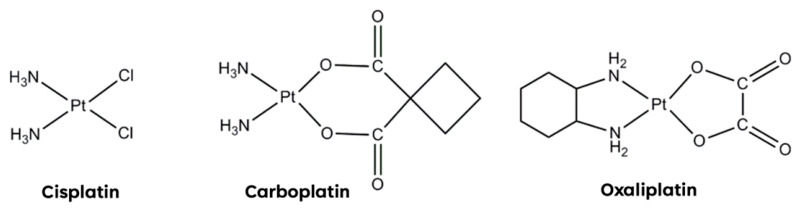
Schematic drawing of cisplatin, carboplatin and oxaliplatin. Cisplatin is a diamminechloroplatinum compound in which the two ammine ligands and the two chloro ligands are oriented in a cis planar configuration around the central platinum ion. Carboplatin contains a platinum atom complexed with two ammine ligands and a cyclobutane-dicarboxyl residue. Oxaliplatin is a complex in which the platinum atom is complexed with 1,2-diaminocyclohexane and an oxalate ligand.

Transition metal complexes find numerous applications in medicine, especially as antitumor agents. Metal-based drugs became effective anti-cancer drugs between the late 1960s and the early 1970s thanks to Rosenberg, who discovered the anti-cancer activity of Pt-based compounds in 1969 [8]. The first Pt-based anti-tumor drug used in clinics was cisplatin (cis-diamminedichloro-Pt(II), CDDP) (Figure 1) [8,9]. Since its first introduction, CDDP has had a major impact on the outcome of a great number of solid tumors, including testicular and ovarian carcinomas, lymphoma, melanoma, and neuroblastoma [10]. However, its use is largely limited by several severe side effects, such as systemic toxicity [11]. An array of CDDP derivatives was developed to enhance therapeutic efficacy and balance CDDP’s adverse effects. These compounds are referred to as second- and third-generation derivatives. Carboplatin (diamminecyclobutane-1,1-dicarboxylate-Pt(II)) [12] and oxaliplatin ((R,R)-1,2-cyclohexanediamine(ethanedioate-O,O)-Pt(II)) [13] are the most used CDDP analogues in clinical practice and are effective for cancers that respond poorly to CDDP (Figure 1). While carboplatin is primarily used to treat ovarian, lung, bladder, head and neck cancers [14], oxaliplatin is employed in the treatment of colorectal carcinoma [15], and is the last platinum-based agent to be approved worldwide.

**Figure 2:**
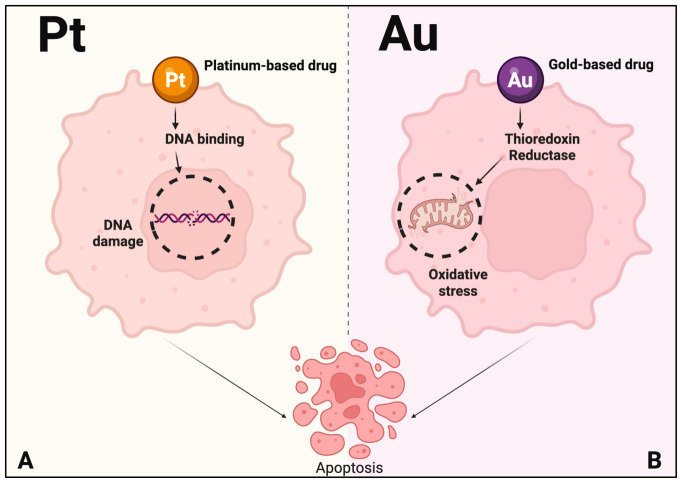
Schematic representation of the principal mechanism of action of platinum- (**A**) and gold-based (**B**) anticancer drugs. The dashed circles highlight the primary intracellular targets involved in their cytotoxic activity. Platinum compounds mainly induce DNA damage through the formation of DNA (in nucleus and mitochondrial) adducts and crosslinks, ultimately triggering apoptosis; whereas gold compounds predominantly target mitochondrial and redox-regulating proteins, leading to oxidative stress, mitochondrial dysfunction, and apoptotic cell death. The illustration is intended as a simplified overview of the major mechanisms and does not exclude the contribution of additional intracellular targets. Created in BioRender. Giorgio, A. (2026) https://BioRender.com/o4990es.

The cytotoxicity of CDDP and its second- and third-generation derivatives is due to the formation of DNA lesions at the double-helix level that interfere with transcription, resulting in cellular apoptosis (Figure 2A). Although nuclear DNA is considered the primary pharmacological target of platinum drugs, increasing evidence suggests that mitochondria DNA damage may also contribute to their cytotoxic activity and apoptosis induction [16]. A crucial role in this process is played by reactive Pt-species that form upon chloride replacement, obtaining DNA crosslinks [17]. Nevertheless, nucleobases are not the only target of Pt compounds, and other biomolecules are likely to be involved in their biological activity. Moreover, also copper transporter (CTR1) was identified as a mediator of CDDP uptake [18]. In this respect, considerable interest has been devoted to the interaction of these complexes with sulfur-containing proteins, leading to nephrotoxicity [11]. Despite their successful applications in clinics, CDDP and other Pt(II) compounds developed in the last few years are limited in efficacy because of their severe toxicity and intrinsic or acquired resistance [19,20].

Following the development of Pt(II) complexes [21], several Pt(IV)-based drugs were developed [22]. These compounds are considered “prodrugs”, since the intracellular reduction of Pt(IV) to Pt(II) leads to the release of the active cytotoxic species [23,24]. Although Pt(II)- and Pt(IV)-based compounds share the same metal center, their pharmacological behavior is fundamentally different. Pt(II) complexes are intrinsically active species that exert their cytotoxic effects through direct interactions with biological targets, primarily DNA, whereas Pt(IV) complexes behave as redox-activated prodrugs and require intracellular reduction to generate the corresponding active Pt(II) species. Consequently, while structural modifications of Pt(II) complexes mainly affect delivery, uptake, and selectivity, functionalization of Pt(IV) systems can additionally influence prodrug stability, reduction kinetics, activation pathways, and the release of coordinated ligands. Pt(IV)-complexes have several potential advantages over their Pt(II) analogues. Specifically, they are less reactive and thus undergo fewer reactions on the way to the tumor, resulting in reduced side effects [25]. They also offer greater structural variability than Pt(II) analogues, allowing optimization of chemical and physical properties. Furthermore, while CDDP and its derivatives are generally administered to patients intravenously, Pt(IV)-based drugs can be taken orally due to greater stability in the gastrointestinal tract [26,27]. Among these, Sartraplatin (cis,trans,cis-ammine(cyclohexylamine)diacetatodichloroPt(IV)) has reached phase III clinical trials for hormone-refractory prostate cancer [28].

The success and limitations of Pt-based drugs [29,30,31,32] are driving forces for the development of new compounds based on different metals for use in the treatment of cancer [33]. In recent years, the field of medicinal inorganic chemistry research has seen a progressive development of other anticancer compounds based on other transition metals, including ruthenium [34], palladium [35], cobalt [36], copper [37], gold [38], and many others [39].

Gold compounds have a long-standing history in traditional medicine. During the early stages of modern pharmacology, gold compounds were tested as anti-infective and anti-tubercular agents [40,41]. Nowadays, Au compounds have limited medical application and are used exclusively for the treatment of severe rheumatoid arthritis, although their activity is associated with numerous cardiac side effects [42,43]. Several attempts to prepare and evaluate new gold compounds, either Au(I) or Au(III), as antitumor agents have been reported [44,45,46,47,48,49,50,51]. The chemistry of Au features some unique aspects, mostly owing to Au’s electronic properties. A rich redox chemistry is associated with its three main oxidation states: Au(0), Au(I), and Au(III). In turn, redox changes are strictly linked to changes in the coordination sphere with a frequent switch from square planar coordination of Au(III) to linear di-coordinated Au(I) complexes [42,52].

Au(I) is a soft cation with a preference for soft ligands. The most important Au(I) compounds for medicinal purposes are thiolates and phosphines, mainly di-coordinated [53]. Auranofin is probably the most famous complex of this class [43]. Notwithstanding, Au(I) complexes do have striking cytotoxicity against haemolymphatic cancers in vitro, and almost all show severe cardiac threats and hence have been rejected for clinical trials.

Au(III) is a borderline cation, showing a preference not only for soft ligands but also for nitrogen donors. Au(III) compounds may be divided into the four following classes: (a) classical mononuclear Au(III) complexes [54,55]; (b) Au(III) porphyrins [56,57]; (c) organogold(III) compounds [58,59,60]; (d) dinuclear Au(III) complexes [61]. Au(III) compounds are considered “prodrugs” and undergo rapid metabolism in vivo. A vast array of Au(III) complexes has potential anticancer activity [62]. These compounds, which show high chemical stability, possess relevant antiproliferative properties in vitro and may be considered excellent candidates for further pharmacological evaluation.

Gold-based drugs mainly act through the modification of selected enzymes [63], which consequently lose their function [62,64,65]. The mechanism of action of Au(I) thiolate and phosphine drugs has been deeply investigated [66]. About Au(III) complexes, generally, they behave as strong oxidizing agents; hence, it is commonly believed that they are quickly reduced to Au(I) compounds or to colloidal Au by low molecular weight biomolecules and by protein residue side-chains [52]. Anyway, different reports have identified thioredoxin reductase (TrxR) as a reliable target for anticancer Au compounds [63]. TrxR is a selenoenzyme critically involved in the regulation of the intracellular redox state and mitochondrial functions [62]. The key event of the mechanism of gold compounds would be the direct, strong inhibition of TrxR that leads to the opening of the mitochondrial pore, likely inducing cytochrome c release and apoptosis (Figure 2B).

The molecular mechanism underlying TrxR inhibition has been further explored by using Au(I) N-heterocyclic carbene (NHC) compounds [67] to clarify the role of direct gold-selenocysteine coordination in enzyme inactivation. Overall, the chemistry of gold complexes in their two oxidation states was investigated in depth, as well as their behavior in solution and interactions with proteins, which play a key role in their biological activity [42,62,63,68,69].

Besides Au(I) and Au(III), the metallic Au(0) state may also play a relevant role in the biological fate of gold-based drugs. Following intracellular reduction, Au(III) complexes can generate Au(I) intermediates and, under strongly reducing conditions, further evolve into elemental gold species, including colloidal gold or gold nanoclusters [52]. The formation of Au(0) is generally regarded as a deactivation pathway because metallic gold lacks the coordination reactivity required for interaction with biological targets. However, Au(0) species may contribute to prolonged tissue retention, while their biodistribution, persistence, and excretion remain only partially understood.

### 1.2. Peptides as Drug Delivery Systems

Natural and synthetic peptides play a variety of biological roles. They act as hormones, inhibitors, biological regulators, and so on. Peptides play essential roles also in medicinal applications, such as therapeutic agents and diagnostic tracers. Their advantages include biocompatibility, low cost, chemical diversity, high affinity, low immunogenicity, and simple synthesis. For these properties, peptides outperform small molecules and large biomolecules, such as monoclonal antibodies, in the biomedicine field [70].

Recently, peptide drug development has made great progress thanks to new production, modification, and advanced analytical technologies. To date, more than 80 therapeutic peptides have reached the global market, and hundreds of them are undergoing preclinical studies and clinical development [70,71]. These drugs are employed in a large range of diseases, such as diabetes mellitus, cardiovascular, gastrointestinal and infectious diseases, cancer, as well as vaccines. Peptides are also considered excellent candidates for drug delivery into cells [72,73,74]; some of them can cross the blood–brain barrier (BBB) [75,76], others can be used to functionalize larger systems, such as nanoparticles [77,78,79,80,81,82,83], liposomes [84,85], and different macroaggregates with applications in cancer therapy and diagnosis. Depending on their biological function, peptides employed in drug delivery can be broadly classified into different categories. Receptor-targeting peptides (RTPs) exploit the overexpression of specific receptors on cancer cells or tumor-associated vasculature to achieve preferential tumor accumulation and internalization [86,87]. Cell-penetrating peptides (CPPs) facilitate cellular uptake through membrane translocation or endocytic pathways and can enhance intracellular accumulation of the conjugated cargo [88]. Organelle-targeting peptides are designed to direct therapeutic agents toward specific intracellular compartments, such as the nucleus (nuclear localization signal, NLSs) sequences or mitochondria (mitochondria-targeting peptides, MTPs), thereby improving target engagement and biological efficacy [89,90,91]. These approaches are not mutually exclusive and can be combined within the same construct to simultaneously promote tumor recognition, cellular uptake, and subcellular localization.

**Figure 3:**
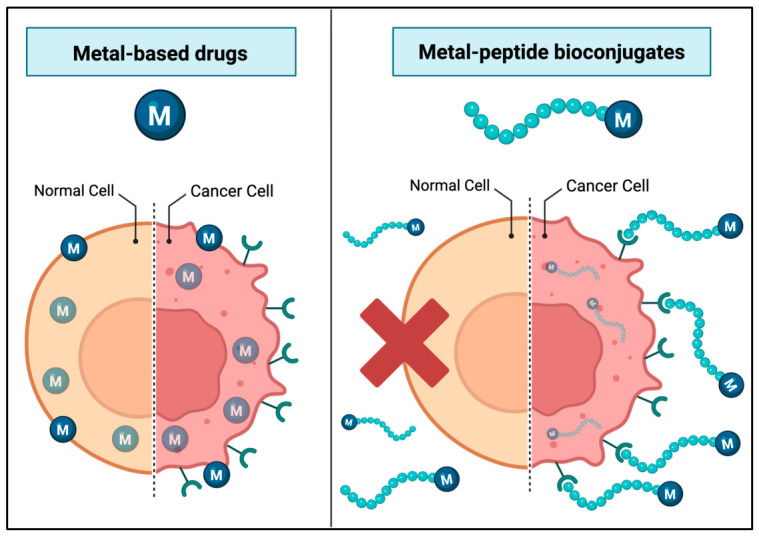
Schematic representation of the enhanced selectivity achieved by metal-peptide bioconjugates compared with unconjugated metal-based drugs. Peptide conjugation may promote several uptakes in cancer cells through different mechanisms, including receptor-mediated recognition and internalization, membrane translocation. In RTPs, the corresponding receptors may also be present in normal cells but are often overexpressed in cancer cells, resulting in preferential accumulation of the bioconjugates at the tumor site. The intracellular localization shown in the cancer cell represents the final outcome of peptide-assisted delivery and does not depict a single universal uptake pathway. The figure is intended as a simplified conceptual overview of the different strategies used to improve selectivity and intracellular delivery of metal-based drugs. Created in BioRender. Giorgio, A. (2026) https://BioRender.com/pgr1jfu.

Recently, peptides achieved great success in drug delivery systems of contrast agents for tumor imaging [92,93] and/or metal-based anticancer drugs [94], reducing the side effects of non-selective metal that also kill normal cells (Figure 3). Furthermore, bispecific radioligands (BRLs) containing two distinct peptide sequences have also emerged; they can exploit a dual targeting capacity and therefore increase the specific uptake of RPs for diagnostic and therapeutic applications in nuclear medicine [95]. In metal-peptide anticancer drugs, peptide moieties can be linked to the metal center, obtaining a wide variety of bioconjugates [96].

In this review, the attention is focused on peptide-based metallo-drugs containing square-planar Pt(II) and linear Au(I) complexes and their higher-valent counterparts, octahedral Pt(IV) and square-planar Au(III) compounds. Directly reactive species, such as Pt(II) and Au(I), exert their therapeutic effects through immediate coordination to biomolecular targets, including DNA in the case of platinum and thiol- or selenol-containing proteins in the case of gold. In these systems, the peptide primarily acts by modulating delivery, selectivity, and subcellular distribution, while preserving the intrinsic reactivity of the metal center. By contrast, Pt(IV) and Au(III) complexes are commonly conceived as prodrugs, relying on redox activation within the cellular environment to generate the corresponding lower-valent and therapeutically active species. In these cases, peptide conjugation serves a dual purpose: improving pharmacokinetic properties and contributing to controlled activation by influencing reduction kinetics and intracellular localization. However, the success of this strategy critically depends on achieving a delicate balance between stability and lability of bioconjugates. Premature reduction can undermine targeting and selectivity, whereas excessive kinetic inertness may limit activation and therapeutic efficacy. Notably, peptide conjugation does not uniformly favor one function over the other. While peptides can enhance uptake and selectivity for both directly reactive and prodrug systems, they may also decouple delivery from biological action. For directly reactive complexes, steric and electronic effects introduced by peptide attachment sometimes can attenuate target engagement, whereas for prodrug systems, interactions with biomolecules may influence metal redox state. Overall, understanding and exploiting the interplay between metal-centered reactivity and peptide-mediated delivery is essential for the rational design of metal-peptide bioconjugates.

### 1.3. Platinum and Gold Complexes: Coordination Chemistry Perspective

Pt(II) complexes remain the cornerstone of metal-based chemotherapy. From a chemical standpoint, Pt(II) has a d^8^ electronic configuration and exhibits a square planar coordination geometry. Pt(II) centers are typically linked to peptides via substitution of labile ligands or through pendant functional groups on spectator ligands, preserving the square-planar geometry required for DNA interaction.

Pt(IV) complexes were widely explored as prodrug candidates designed to overcome the limitations of Pt(II)-based chemotherapy. Pt(IV) has a d^6^ electronic configuration, and its octahedral geometry and kinetic inertness allow for functionalization with axial ligands, making Pt(IV) particularly attractive for peptide conjugation.

Gold(I) is a d^10^ closed-shell transition metal, with three principal coordination geometries: linear two- (by far the most important), trigonal three- or tetrahedral four-coordination.

Gold(III) has a d^8^ closed-shell configuration. Gold(III) complexes have attracted interest in anticancer research due to their formal analogy to square-planar Pt(II) systems and their potential to engage in coordination geometries compatible with peptide conjugation.

Among the numerous metal-based anticancer agents investigated to date, platinum and gold were selected as the focus of this review because they provide the opportunity to compare peptide conjugation strategies in both directly reactive and redox-activated systems. Platinum remains the most clinically successful class of metal-based anticancer drugs, whereas gold compounds have emerged as promising alternatives with distinct mechanisms of action. Moreover, these two metals display an intriguing parallel in their medicinal chemistry: Pt(II) and Au(I) generally represent the biologically active forms that directly interact with cellular targets, while Pt(IV) and Au(III) are commonly developed as prodrugs that require intracellular reduction to generate the active species. In addition, Pt(II) and Au(III) share a square-planar coordination geometry, making them particularly suitable for a comparative analysis of how peptide conjugation influences stability, targeting, cellular uptake, and activation pathways.

## 2. Materials and Methods

An extensive literature search was conducted across different databases, including PubMed, Google Scholar, Scopus, EMBASE, and Web of Science, focusing on the most recent publications from the past two decades. The search strategy incorporated a structured combination of keywords such as “ metal peptide conjugates”, “gold peptide conjugates”, “platinum peptide conjugates”, and “cancer therapy”. The screening process was performed independently by two authors. Search terms were adjusted as necessary for each database, and Boolean operators (AND, OR) were applied to refine results. Additionally, the reference lists of the selected studies were examined manually to identify further relevant literature.

Following the literature screening process, a total of 13 studies on Pt(II)-peptide conjugates, 16 studies on Pt(IV)-peptide conjugates, 7 studies on Au(I)-peptide conjugates, and 2 studies on Au(III)-peptide conjugates, were selected for detailed analysis and discussion. The selected publications were chosen based on their relevance to the scope of this review, with particular emphasis on molecular design, conjugation strategies, biological activity, mechanism of action, and potential applications in cancer therapy.

## 3. Platinum-Peptide Conjugates

### 3.1. Pt(II)-Peptide Conjugates

**Figure 4:**
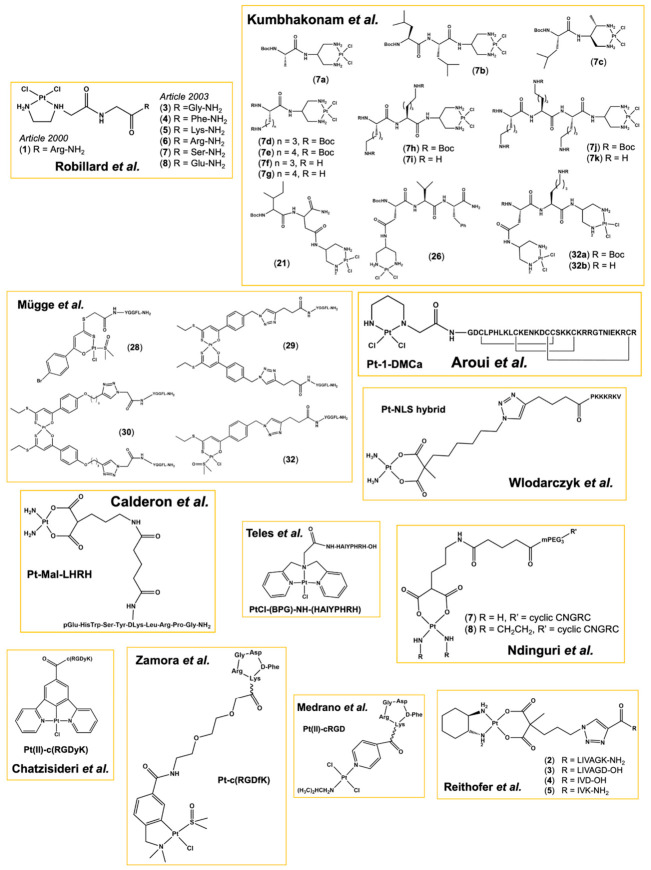
Structure of all discussed Pt(II)-peptide bioconjugates [97,98,99,100,101,102,103,104,105,106,107,108,109].

Among the Pt(II) complexes, CDDP and its analogues exerting their anticancer activity primarily through DNA binding and crosslink formation. In this context, peptide conjugation has been extensively investigated as a strategy to enhance cellular uptake, improve tumor selectivity (by recognizing tumor overexpressed receptors), reduce side-effects, and modulate pharmacokinetic profiles of Pt(II) drugs. Also, peptides have been employed to improve the solubility of complexes and DNA selectivity. Pt(II)-peptide conjugates exemplify the challenges of combining peptide-mediated targeting while preserving DNA-reactive metal centers. Although selective uptake can be achieved, maintaining the fine balance between stability, reactivity, and intracellular accessibility remains a critical hurdle for the successful development of targeted Pt(II)-based therapeutics. Various studies present in the literature are reported below (Table 1 and Figure 4).

Robillard and coworkers reported the potentially active trimeric Arg-containing Pt(II) complex **1** [97]. The target molecule includes an Arg-Gly dipeptide tethered to an ethylenediamine moiety as a Pt-chelating ligand. It involves the use of N-2-aminoethyl-glycine (AEG). A few years later, same authors published other studies [98,110] aimed to evaluate whether further peptide-tethered Pt(II) complexes modify the intensity and sequence specificity of DNA damage compared to CDDP and the related compound Pt(en)Cl_2_ (en = ethylenediamine) [98]. Six bioconjugates (**3**–**8**), differing only in their pendant amino acid (Gly, Phe, Lys, Arg, Ser or Glu), were synthesized. DNA damage was assessed in pUC19 plasmid DNA and in HeLa cells. All bioconjugates exhibited DNA sequence specificity similar to CDDP and Pt(en)Cl_2_, with preferential damage at sites containing consecutive guanine residues. In pUC19 plasmid DNA, the Gly- and Phe-tethered complexes produced the most serious damage, followed by Lys, Arg, Ser, and Glu derivatives. In HeLa cells, DNA damage levels were generally lower than those induced by CDDP, with the Lys- and Arg-tethered complexes showing the highest cellular activity, while Gly- and Phe- complexes displayed a marked reduction in effectiveness compared to plasmid DNA. The nature of the appended amino acid affects Pt reactivity and cellular DNA damage, likely through differences in charge and polarity. These findings indicate that short peptide conjugation alone is insufficient to enhance DNA targeting, and an appropriate peptide design is still required for improving Pt-based drug delivery and activity.

Kumbhakonam et al. developed a modular synthetic strategy for the site-specific incorporation of Pt(II) centers into peptides using Ser- and Thr-derived diamines [99]. A key advantage of the methodology used offers high versatility, allowing platinum centers to be introduced at different positions within a peptide sequence, including the N-terminus, C-terminus, side-chain functionalities, or at multiple sites simultaneously. Authors synthesized a library of Pt-peptide conjugates containing hydrophobic residues and positively charged Lys- and ornithine-rich sequences (**7a**–**k**, **21**, **26**). In addition, multinuclear platinum conjugates bearing two Pt(II) centers were successfully prepared (**32a**–**b**). Conjugates containing positively charged Lys side chains exhibited significantly stronger DNA binding than their neutral analogues, highlighting the contribution of electrostatic interactions with the DNA phosphate backbone. Cytotoxicity studies against SiHa cervical cancer cells revealed a dose-dependent antiproliferative effect for all Pt-containing conjugates. The most active compounds were the Lys-rich derivatives.

Mügge et al. reported the synthetic strategies for the preparation of Pt(II)-peptide conjugates containing β-hydroxydithiocinnamic ester ligands as versatile (O,S)-chelating frameworks [100]. Using Leu5-enkephalin (YGGFL) as a model peptide, authors compared two major bioconjugation approaches, amide-bond formation and click chemistry, while evaluating the influence of the synthetic sequence on the final products. The amide-coupling route proved particularly effective when peptide conjugation preceded metal coordination, leading to the successful isolation of the peptide-functionalized Pt(II) monochelate (**28**) in good yield. The reaction between an azide-functionalized Pt bischelate and an alkyne-modified peptide afforded conjugate (**29**) with comparatively high conversion and fewer by-products, demonstrating the synthetic advantages of incorporating the azide functionality within the metal-containing fragment. In contrast, the complementary strategy employing an alkyne-bearing Pt complex generated conjugate (**30**) in lower yield due to side reactions associated with the alkyne-containing β-hydroxydithiocinnamic ester framework. To access a mononuclear peptide-Pt(II) conjugate through click chemistry, the authors first synthesized the metal-free triazole-linked precursor and subsequently introduced the Pt(II) center, obtaining complex (**32**) while retaining the chlorido and DMSO ligands.

Aroui et al. developed a novel Pt-based anticancer conjugate, **Pt-1-DMCa**, by linking a platinum-chelating moiety (MBL-III-7) to D-maurocalcine (D-MCa, GDCLPHLKLCKENKDCCSKKCKRRGTNIEKRCR), a protease-resistant CPP derived from the scorpion toxin maurocalcine [101]. Biological assays demonstrated that **Pt-1-DMCa** exhibited, particularly at low concentrations, an IC_50_ approximately two-fold lower than that of CDDP. Importantly, the conjugate exhibited reduced toxicity toward normal astrocytes, suggesting improved selectivity for malignant cells. Moreover, **Pt-1-DMCa** induced substantial DNA damage and increased phosphorylation of p53 and histone H2A.X. Further investigation demonstrated that **Pt-1-DMCa** activated both intrinsic and extrinsic apoptotic pathways. In addition, **Pt-1-DMCa** induced a marked accumulation of reactive oxygen species, which contributed to DNA damage and apoptosis and, unlike CDDP, it also inhibited AKT and ERK phosphorylation.

In their study, Wlodarczyk and colleagues developed a Pt(II)-hybrid system containing an NLS peptide that delivers Pt(II) directly to the nucleus and overcomes Pt resistance in cancer cells [102]. The SV40 large T antigen-derived NLS peptide (PKKKRKV) was synthesized, and the carboplatin-like Pt(II) complex was coupled to the N-terminus of the peptide by click chemistry, affording the **Pt-NLS hybrid**. Cellular and nuclear uptake were demonstrated in ovarian cancer-derived cell lines with different platinum resistance (A2780, CP70, TOV-21G, SKOV3, ES-2, OV-90). Viability assay proved high cytotoxicity of **Pt-NLS hybrid**, significantly increased with respect to carboplatin, regardless of the cell line tested. The greatest cytotoxic effect was observed in A2780 cells, where viability was decreased by 60%, whereas treatment with carboplatin at the same concentration decreased viability by 25% only. In the other cell types, **Pt-NLS hybrid** also had a notably larger effect on cell viability than carboplatin. Overall results indicate that the **Pt-NLS hybrid** delivers Pt(II) into the nucleus, markedly enhancing cytotoxicity and overcoming resistance.

Calderon et al. developed a targeted Pt-based chemotherapeutic agent, **Pt-Mal-LHRH**, by conjugating carboplatin to the luteinizing hormone-releasing hormone (LHRH, pGlu-His-Trp-Ser-Tyr-DLys-Leu-Arg-Pro-Gly) peptide through a malonate linker [103]. LHRH receptors are overexpressed in several cancers, including breast cancer, while showing limited expression in normal tissues. Biological evaluation was performed primarily in breast cancer cell lines (murine 4T1 and human MDA-MB-231). The conjugate exhibited significantly enhanced cytotoxicity and selectivity toward cancer cells, while showing lower toxicity against normal fibroblasts (3T3). **Pt-Mal-LHRH** cellular uptake achieved approximately 20-fold higher cellular platinum uptake than carboplatin in the 4T1 cell line, consistent with receptor-mediated internalization through the LHRH receptor. Furthermore, competition experiments with free LHRH confirmed the involvement of receptor-specific uptake pathways. The antitumor efficacy of **Pt-Mal-LHRH** was subsequently evaluated in vivo using an orthotopic 4T1 breast cancer mouse model. Mice treated with **Pt-Mal-LHRH** displayed a significant reduction in tumor volume compared with untreated controls and carboplatin-treated animals. In addition, metastatic colonization of the lungs was markedly reduced, indicating that the conjugate effectively suppressed both primary tumor growth and metastatic dissemination.

The study of Teles and colleagues was focused on a Pt(II)-peptide conjugate targeting the transferrin receptor (TfrR), to improve the selectivity of Pt-based anticancer agents [104]. A new Pt(II) complex, PtCl-(BPG) (BPG = bis(2-pyridylmethyl)glycine), was successfully conjugated to the TfrR binding peptide HAIYPHRH. The conjugate PtCl-(BPG)-NH-(HAIYPHRH) (**1**) structural characterization revealed a stable coordination of Pt(II) to the tridentate nitrogen ligand, to one of which the peptide is conjugated via a carboxylic acid. Biological studies showed that the conjugate binds strongly to serum albumin and covalently interacts with DNA without inducing strand breaks. In vitro antiproliferative assays demonstrated growth inhibition in renal cancer cells (786-0). However, peptide conjugation did not enhance cytotoxic activity compared to the precursor Pt(II) complex, bringing out the limitations and challenges associated with the development of Pt(II)-peptide conjugates targeting TfrR.

The work of Ndinguri and coworkers aimed to improve the selectivity and potency of Pt(II)-based chemotherapy by covalently attaching a CD13-targeting peptide (Asn-Gly-Arg, NGR motif) to a platinum carrier, thereby creating a low-molecular-weight, water-soluble Pt-peptide conjugate that could be preferentially taken up by CD13-positive prostate-cancer cells (PC13) [105]. Authors prepared cyclic mPEG-CNGRC-Pt (**7**) and cyclic mPEG-CNGRC-Pten (**8**) conjugates in which the NGR sequence was conjugated to a malonoyl-based linker that bound a carboplatin-like platinum center; the mPEG was inserted as a linker for non-immunogenicity and to improve solubility. Cellular uptake studies with PC-3 cells showed that the **7** and **8** conjugates delivered up to 12- and 3-fold more to the cells than untargeted carboplatin, respectively. Cytotoxicity assays demonstrated that bioconjugates were markedly more effective than carboplatin alone and induced apoptosis at concentrations lower than the parent drug. Authors concluded that the NGR-guided Pt conjugates achieve tumor-cell-specific delivery and significantly enhance anticancer activity while maintaining low toxicity toward normal cells.

Chatzisideri et al. designated **Pt(II)-c(RGDyK)** by coupling a cyclometalated [N,C,N]-Pt(II) complex to the cyclic peptide c(RGDyK) through a stable amide bond [106]. The cyclometalated platinum complex was selected for its intrinsic luminescent properties, enabling real-time monitoring of cellular uptake, and its ability to generate reactive oxygen species upon light irradiation, providing a theranostic platform for targeted photodynamic therapy. The biological evaluation of the conjugate demonstrated efficient and selective uptake in α_V_β_3_-positive U87 glioblastoma cells. Confocal microscopy studies revealed significantly stronger intracellular fluorescence for **Pt(II)-c(RGDyK)** compared with the non-targeted platinum precursor, while uptake was markedly reduced in HeLa cells expressing lower levels of α_V_β_3_ integrins. In antiproliferative assays, the conjugate exhibited moderate cytostatic activity across several integrin-expressing cancer cell lines, with enhanced growth inhibition observed in SKOV-3 and MDA-MB-231 cancer cells compared with the unconjugated platinum complex. Photophysical studies demonstrated efficient singlet oxygen generation upon blue-light irradiation, supporting its application as a photosensitizer. Consistently, photodynamic therapy experiments in AY27 bladder cancer cells showed a significant reduction in cell viability following light exposure, with the conjugate producing greater antitumor effects than the parent platinum complex.

Zamora and coworkers synthesized a novel bioconjugate linking a cyclometalated Pt(II) complex (PtCl(dmba)(dmso)) (dmba = C,N-dimethylbenzylamine), which exhibits antiangiogenic and antitumor activity, to a cyclic RGD peptide (c(RGDfK)) targeting the α_V_β_3_ integrin receptor, overexpressed in several types of cancer, tumor-associated vasculature, and invasive tumor fronts, and focused on antiangiogenic properties [107]. The bioconjugate **Pt-c(RGDfK)** was synthesized and modified via the lysine residue to incorporate a PEG spacer, which then reacted with the activated Pt complex. Cytotoxicity was determined by the MTT assay across several human cancer cell lines, showing different levels of integrin expression, and antiangiogenic potency was assessed using the HUVEC tube formation assay. The parent Pt complex showed sub-micromolar cytotoxicity against ovarian cancer cells (A2780) but was inactive against other tested cell lines (SK-MEL-28, MDA-MB-231, CAPAN-1, and HUVEC). Conjugation to c-(RGDfK), even if abolished the antitumor activity, allows for preserving the antiangiogenic potency, inhibiting HUVEC tube formation without cytotoxicity. Furthermore, the bioconjugate induced a modest reduction in the number of adherent cells in both SK-MEL-28 and MDA-MB-231 cell lines, in accordance with the presence of RGD peptide.

Remaining in the context of RGD peptides, Medrano and coworkers investigated non-conventional trans-Pt(II) complexes functionalized with the c-(RGDfK) peptide (cRGD) [108]. The peptide was covalently attached through a 4-picolinic acid spectator ligand, yielding a **Pt(II)-cRGD** bioconjugate fully characterized by spectroscopic techniques, including ^195^Pt NMR. Conjugation of the Pt(II) core to the cRGD peptide did not lead to a significant enhancement of cytotoxicity when compared with the corresponding non-conjugated Pt(II) precursor. In most cancer cell lines tested (MDA-MB-231, MCF7, A2780, SKOV3 and A549) expressing integrin at different levels, peptide functionalization even resulted in reduced antiproliferative activity. However, in endothelial HUVEC cells, which overexpress the α_V_β_3_ integrin receptor, the **Pt(II)-cRGD** conjugate retained measurable cytotoxicity. Importantly, cellular uptake studies revealed that peptide conjugation substantially increased intracellular platinum accumulation measured by ICP-MS, particularly in integrin-positive cells. Competitive blocking experiments on HUVEC cells with an excess of free cRGD partially reduced platinum uptake, supporting the involvement of α_V_β_3_-mediated recognition.

The study of Reithofer and coworkers aimed to develop a biocompatible platform by conjugating oxaliplatin derivatives to self-assembling short aliphatic peptides that were functionalized at their N-terminus with the oxaliplatin-derived moiety using click chemistry (**2**–**5**) [109]. While the Pt-peptide conjugates alone generally did not self-assemble, short peptides were co-assembled with the parent peptide to form hybrid injectable hydrogels with high drug loadings (up to 40 wt%). Hybrid hydrogels preserved the characteristic nanostructured morphology of the parent peptide scaffold and enabled sustained release of the Pt drug. In vitro, bioconjugates displayed micromolar cytotoxicity across several cancer cell lines (HeLa, SW480, 4T1), induced G2/M cell cycle arrest, activated caspase-dependent apoptosis, and were capable of DNA platination, albeit to a lower extent than free oxaliplatin. In vivo, localized administration of the oxaliplatin-peptide hydrogel significantly inhibited tumor growth, while exhibiting markedly reduced systemic toxicity compared to free oxaliplatin. Biodistribution studies revealed enhanced Pt accumulation at the tumor site and lower Pt levels in the liver and kidneys, consistent with a controlled and localized release profile. This work demonstrates that self-assembling short peptides can serve as effective carriers for Pt-based drugs, improving tumor retention and tolerability without abolishing anticancer activity.

As can be seen from the collected studies, carboplatin and oxaliplatin derivatives are often used as functional complexes by modifying one of their ligands. The literature on Pt(II)-peptide conjugates shows that peptide attachment can influence platinum reactivity, cellular uptake, and biological behavior, although it does not always lead to improved anticancer efficacy. Advanced design demonstrates that functional peptides, such as RTPs or CPPs, can enhance intracellular accumulation, subcellular localization, and help to overcome platinum resistance. The data reported indicated that increased uptake may not necessarily translate into proportional gains in cytotoxicity, revealing a lack of direct correlation between delivery and pharmacological effect. Overall, Pt(II)-peptide conjugation emerges as a versatile but challenging strategy whose success critically depends on rationally designed peptide, linker selection and peptide attachment.

### 3.2. Pt(IV)-Peptide Conjugates

The anticancer activity of Pt(IV) complexes critically depends on their intracellular reduction to the corresponding Pt(II) species. This activation process is generally promoted by biological reducing agents, including glutathione, ascorbate, Cys, and other redox-active biomolecules, although the relative contribution of each reductant may vary depending on the cellular context and the structure of the complex [111,112]. Upon reduction, the octahedral Pt(IV) center undergoes conversion to a square-planar Pt(II) species, accompanied by the release of the two axial ligands [111]. Consequently, the reduction kinetics of Pt(IV) prodrugs play a central role in determining their therapeutic efficacy. Excessively rapid reduction may lead to premature activation and loss of selectivity, whereas overly stable Pt(IV) complexes may fail to generate sufficient amounts of the active Pt(II) drug within cancer cells. Therefore, an optimal balance between extracellular stability and intracellular activation is required [112,113].

Axial functionalization represents one of the most attractive features of Pt(IV) chemistry. Unlike Pt(II) complexes, which offer limited opportunities for derivatization without affecting their biological activity, Pt(IV) complexes possess two axial coordination sites that can be exploited to modulate physicochemical and biological properties [111,114]. The introduction of peptides or other targeting moieties in axial positions may improve water solubility, biodistribution, and reduction kinetics [112,113,114]. Furthermore, because axial ligands are typically released during reduction, they may also function as bioactive payloads, enabling the design of dual-action prodrugs that simultaneously generate a cytotoxic Pt(II) species and release a second therapeutic or targeting agent [111,114]. Notably, in peptide-conjugated Pt(IV) systems, therapeutic performance is not dictated solely by the reduction potential of the metal center but also by the interplay between reduction kinetics, peptide-mediated targeting, intracellular trafficking, and the biological fate of the released axial ligands.

**Figure 5:**
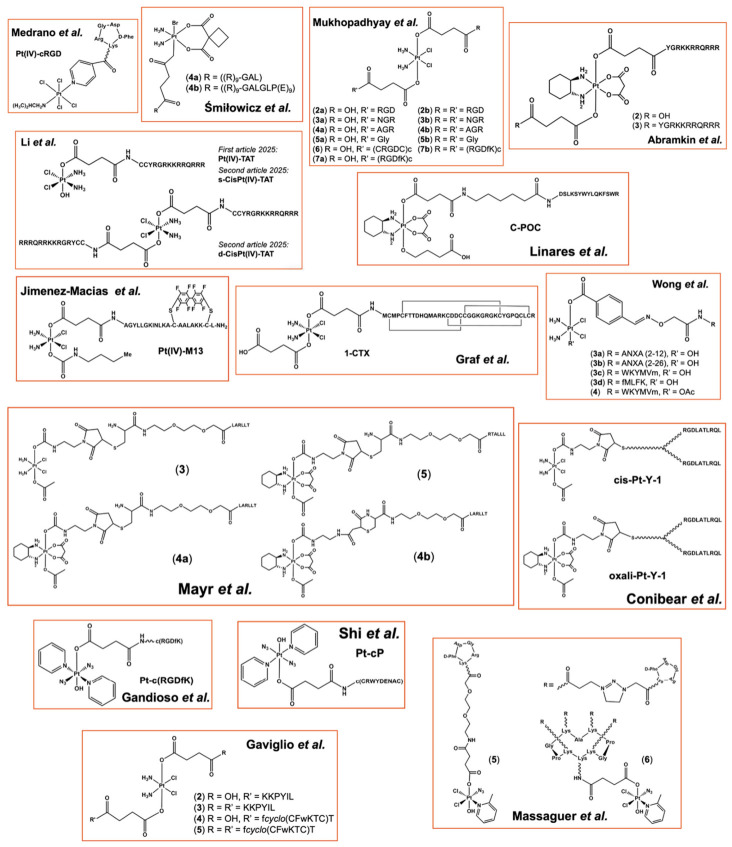
Structure of all discussed Pt(IV)-peptide bioconjugates [108,115,116,117,118,119,120,121,122,123,124,125,126,127,128,129].

Numerous Pt(IV)-peptide conjugates were developed in which peptides are covalently attached through axial carboxylate or amide linkages. Several studies reported in the literature are shared below (Table 2 and Figure 5).

Medrano and colleagues explored the conjugation of analogous trans-Pt(IV) complexes containing isopropylamine and pyridine ligands to the cRGDfK peptide (**Pt(IV)-cRGD**) [108]. The rationale was that intracellular reduction of this bioconjugate could release an active Pt(II) species while maintaining peptide-driven selectivity. Although the formation of the **Pt(IV)-cRGD** was supported by mass spectrometry and chromatographic data, ^195^Pt NMR experiments revealed that the Pt(IV) conjugate underwent rapid reduction to its Pt(II) counterpart in solution, indicating insufficient redox stability of the construct under the investigated conditions. Consequently, biological evaluation of the bioconjugate was not pursued.

Śmiłowicz and Metzler-Nolte developed a synthetic strategy for the conjugation of Pt(IV) with the enhanced cellular uptake provided by polyarginine-containing peptides, CPPs able to efficiently cross cellular membranes [115]. Two peptide vectors were investigated: a positively charged polyarginine sequence P1 ((R)_9_-GAL) and a modified analogue containing an additional polyglutamic acid segment intended to neutralize the positive charge P2 ((R)_9_-GALGLP(E)_9_), obtaining **4a** and **4b** bioconjugates, respectively. The antiproliferative activity of the Pt(IV) precursors and the resulting bioconjugates was evaluated against different cancer cells (HepG2, MCF-7), and normal human fibroblasts (GM5657T). As expected for Pt(IV) prodrugs exhibited lower cytotoxicity than their Pt(II) counterparts. However, the polyarginine-containing bioconjugate **4a** displayed slightly enhanced activity against HepG2 cells compared with the corresponding unconjugated Pt(IV) complex, indicating that the CPP sequence facilitated cellular uptake. In contrast, bioconjugate **4b**, containing both polyarginine and polyglutamic acid residues, showed virtually no antiproliferative activity toward any of the tested cell lines. The loss of activity was attributed to charge neutralization, which likely reduced membrane penetration and intracellular accumulation.

The study of Mukhopadhyay et al. aimed to develop Pt-based anticancer agents with enhanced selectivity toward tumor-associated vasculature and tumor cells by exploiting peptide ligands that recognize integrins (α_V_β_3_ and α_V_β_5_) and aminopeptidase N (APN or CD13) receptors, both highly expressed in tumor-induced angiogenesis [116]. A series of mono-functionalized (**2a**, **3a**, **6**, **7a**) and di-functionalized (**2b**, **3b**, **7b**) Pt(IV) conjugates were designed in which RGD- or NGR-containing peptides were covalently attached as axial ligands by an amide linkage to a CDDP-derived Pt(IV) scaffold through a succinate group. Both linear (RGD, NGR) and cyclic peptides ((RGDfK)c, (CRGDC)c) were employed to evaluate the impact of the peptide on targeting efficiency. Non-targeting peptide (AGR, **4a** and **4b**) or amino acid (Gly, **5a** and **5b**) conjugates were synthesized as negative controls. The antiproliferative activity of the conjugates was assessed in vitro using primary endothelial cells (BCE, HMVEC, HUVEC) and several tumor cell lines (U87, ASPC1, MES-SA and HeLa) expressing APN and/or α_V_β_3_/α_V_β_5_ integrins. Bioconjugates bearing RGD motifs (**2a**, **2b**, **6**, **7a**, **7b**) exhibited significantly enhanced antiproliferative activity compared to non-targeted Pt(IV) analogues and a potency similar to CDDP in endothelial cells. NGR-containing bioconjugates (**3a**, **3b**) displayed intermediate activity, consistent with their lower affinity for integrin receptors. Nontargeting bioconjugates (**4a**, **4b**, **5a**, **5b**) were an order of magnitude less active. Free peptides did not inhibit cell growth. Competition experiments using free peptides were performed to elucidate the role of integrin-mediated recognition. No major differences were observed between mono- and di-functionalized conjugates, suggesting that a single targeting ligand is sufficient for effective receptor recognition.

In their work, Abramkin and coworkers reported a bioconjugate resulting from the covalent conjugation of the CPP HIV-1 TAT (YGRKKRRQRRR) to a Pt(IV)-derived oxaliplatin prodrug, to achieve an enhanced antiproliferative effect [117]. Both mono-functionalized (**2**) and a di-functionalized (**3**) conjugates displayed low-micromolar IC_50_ values in four human cancer cell lines (CH1, SKOV-3, SW480, A549), markedly superior to the non-targeted carboxylate and amide analogues. Bioconjugate **2** was up to 39-fold more potent than the carboxylate and 8-fold more potent than the amide derivative in CH1 cell lines. In general, all IC_50_ values of bioconjugate **2** were slightly lower in all cell lines compared to those of bioconjugate **3**. TAT-mediated delivery dramatically enhances oxaliplatin’s antiproliferative activity, with the carboxylate derivative **2** showing the greatest potency.

Li et al. developed a novel Pt(IV) prodrug, **Pt(IV)-TAT**, by covalently conjugating a CDDP-derived Pt(IV) precursor to the TAT peptide (CCYRGRKKRRQRRR) containing a NLS [118]. **Pt(IV)-TAT** was evaluated in several tumor cells (4T1 and A549). In 4T1 cells, **Pt(IV)-TAT** displayed cytotoxicity comparable to or greater than that of CDDP. Importantly, the conjugate exhibited substantially lower toxicity toward normal epithelial and hepatic cells, suggesting improved selectivity for malignant cells. **Pt(IV)-TAT** inhibited proliferation, induced apoptosis, and promoted G2-phase cell-cycle arrest. Platinum levels in isolated nuclei were approximately four-fold higher following **Pt(IV)-TAT** treatment, while DNA-associated platinum content was also markedly increased. In vivo studies using a 4T1 tumor-bearing mouse model further demonstrated that **Pt(IV)-TAT** significantly suppressed tumor growth after intravenous administration while producing minimal systemic toxicity. Tumor tissues showed increased platinum accumulation and enhanced apoptosis compared with controls and conventional platinum treatment.

Subsequently, same authors investigated how the degree of peptide functionalization influences its biological performance, synthesizing **d-CisPt(IV)-TAT** containing two TAT peptides attached to the same Pt(IV) scaffold [119]. Results showed that the previous bioconjugate accumulated more effectively in whole cells, nuclei, and genomic DNA than **d-CisPt(IV)-TAT**, indicating that simply increasing the number of targeting peptides does not necessarily improve therapeutic performance.

Linares et al. designed the **C-POC** bioconjugate composed of a CPP (CPP2, DSLKSYWYLQKFSWR) and a Pt(IV) prodrug oxaliplatin-derivative [120]. The cytotoxicity of **C-POC** was assessed in colorectal cancer (CRC) cells (LoVo, HT29, SW620) and organoids (PDOs). The conjugate induced an improved cytotoxic activity against PDOs with respect to oxaliplatin. The biological activity was also evaluated in an in vivo model of CRC obtained by subcutaneous xenotransplantation of PDOs in mice, and the results indicated that oxaliplatin and **C-POC** reduce the cancer progression similarly. Notably, biodistribution studies showed that in tumor microenvironment (TME), the Pt-uptake, compared to that of oxaliplatin, was markedly lowered, and the downregulation of versican, a marker of poor prognosis in CRC, was observed. This latter result was relevant as Pt-based drugs generally promote cancer progression and chemoresistance in noncancerous cells within the TME [130,131,132].

Jimenez-Macias et al. developed a novel Pt(IV)-peptide conjugate, **Pt(IV)-M13**, consisting of a CDDP-derived Pt(IV) prodrug covalently linked to M13 (AGYLLGKINLKACAALAKKCL), a brain-penetrant macrocyclic CPP derived from Transportan 10 [121]. Authors first demonstrated that **Pt(IV)-M13** undergoes intracellular reduction, releasing active CDDP. The conjugate was then evaluated in a panel of glioblastoma cell lines (GBM cell lines G30-LRP, G34-pCDH, GBM-X6, G9-pCDH cell line, and patient-derived cell line BT286), where it exhibited significantly greater cytotoxicity than the unconjugated Pt(IV) prodrug and, in some models, activity approaching that of CDDP. Enhanced biological activity correlated with increased intracellular platinum accumulation, indicating that the peptide improved cellular uptake of the platinum payload. To investigate BBB penetration, the conjugate was tested in a three-dimensional BBB spheroid model. ICP-MS analysis revealed markedly higher platinum uptake for **Pt(IV)-M13** compared with CDDP or Pt(IV) precursor, without compromising barrier integrity. In orthotopic glioblastoma mouse models, intravenous administration of **Pt(IV)-M13** resulted in approximately seven- to eight-fold higher platinum levels in both normal brain tissue and tumors relative to CDDP. Importantly, **Pt(IV)-M13** was well tolerated at doses up to three times higher than the maximum tolerated dose of CDDP. At these elevated doses, the conjugate significantly prolonged survival in tumor-bearing mice and induced enhanced DNA damage within tumors.

Graf et al. developed a Pt(IV)-peptide bioconjugate, **Pt-CTX**, by coupling a CDDP-derived Pt(IV) prodrug to chlorotoxin (CTX, MCMPCFTTDHQMAR), a peptide known for its selective binding to several cancer-associated cell surface receptors, including matrix metalloproteinase-2, chloride ion channels, and annexin A2 [122]. The biological activity of the conjugate was evaluated in different cancer cell lines (HeLa, MCF7 and A549). The **Pt-CTX** conjugate exhibited greater antiproliferative activity than both free CTX and the Pt(IV) precursor complex in all cell lines tested, although it remained less potent than CDDP, as expected for a Pt(IV) prodrug. Notably, the most pronounced targeting effect was observed in HeLa cells, where conjugation to CTX enhanced cytotoxicity by approximately 50-fold relative to the unconjugated Pt(IV) precursor.

Wong and colleagues developed a rationally designed Pt(IV)-based bifunctional prodrug that combines conventional chemotherapy with immunotherapy [123]. The bioconjugate was formed by a peptide that simultaneously targets the formyl-peptide receptors FPR1/2 (highly expressed on immune cells and many metastatic cancers) and acts as an immune adjuvant, and by cytotoxic platinum complex. A Pt(IV)-derived CDDP was synthesized and functionalized with four different FPR1/2-binding peptides (ANXA1 2-12, ANXA1 2-26, WKYMVm, and fMLFK) through chemoselective oxime ligation. In vitro cytotoxicity of bioconjugates **3a**–**d** and **4** was evaluated on three cancer cell lines (U-87MG, MCF-7, MDA-MB-231) and compared with CDDP. Among bioconjugates, the WKYMVm-derived prodrugs **3c** and **4** displayed IC_50_ values comparable to or slightly better than CDDP across all three cell lines. In contrast, the fMLFK conjugate **3d** was essentially non-cytotoxic. The tumoricidal activity of drug-activated peripheral blood mononuclear cells (PBMCs) demonstrated that FPR-targeted bioconjugates **3c** and **4** exhibited greater potency than the positive control CDDP. The precise reason for the slightly enhanced potency of **4** over **3c** is not fully understood. This could be due to a shielding effect against Pt(IV) reduction in the used culture medium containing reducing glutathione. To note, free WKYMVm peptide when administered alone did not demonstrate significant cell-mediated cytotoxicity. Immune-mediated effects were examined by co-culturing treated tumor cells with PBMCs and by measuring pro-inflammatory cytokines release (TNF-α, IFN-γ) Importantly, pretreatment of PBMCs with the bioconjugate **4** markedly increased secretion of both TNF-α and IFN-γ, confirming potent innate immune activation. The present study demonstrates that FPR1/2-targeted Pt(IV) prodrugs can simultaneously deliver CDDP to tumor cells and act as strong immunostimulants, providing a feasible multimodal platform for future immuno-chemotherapeutic agents.

The study of Mayr and coworkers aimed to develop bioconjugates selective for epidermal growth factor receptor (EGFR) [124]. EGFR-binding peptide (LARLLT) was conjugated to Pt(IV) complex to enhance tumor selectivity and cellular uptake in EGFR-overexpressing cancer cells. Maleimide-functionalized Pt(IV) complex, derived from CDDP and oxaliplatin, were synthesized and covalently linked to Cys-containing LARLLT peptides via a miniPEG spacer (**3** and **4a**). A shuffled peptide-based (RTALLL) bioconjugate **5** was employed as a non-targeted control. A side byproduct of **4a** was obtained from an unexpected intramolecular trans-cyclization at the maleimide-Cys linkage (**4b**). The biological activity, such as long-term clonogenic effects, Pt uptake, and EGFR dependency, was assessed across a panel of cancer cell lines with varying EGFR expression levels and diverse sensitivity to EGFR-inhibitory treatment (A431, RUMH, HCC827, and H520). Although the Pt(IV)-peptide conjugates retained their ability to undergo reductive activation towards Pt(II) derivatives, neither cytotoxicity nor cellular platinum accumulation correlated with EGFR expression levels, even if all drug accumulation was markedly increased. The LARLLT-conjugated complexes did not outperform the shuffled peptide control in short-term viability assays, long-term clonogenic experiments, or uptake studies.

Conibear’s group focused on the development of multifunctional peptide-drug conjugates targeting the α_V_β_6_ integrin, frequently overexpressed in cancer cells [125]. Branched Y-shaped scaffold comprising a short peptide linker and two monodisperse PEG27 chains, which provide spatial separation between the targeting ligands, was designed and synthesized. Terminal alkyne groups on the PEG chains provide chemoselective conjugation to two copies of α_V_β_6_ integrin-targeting peptide P1 (RGDLATRLQL) via click chemistry. Additionally, a Cys residue within the Y-scaffold enables site-specific attachment of CDDP- and oxaliplatin-derived Pt(IV) complexes **cis-Pt-Y-1** and **oxali-Pt-Y-1** through thiol-maleimide ligation. The binding specificity and cellular uptake of the resulting constructs were assessed using cell lines (SW480 ITGB6) engineered to express low and high levels of β_6_ integrin. Uptake studies demonstrate that **oxali-Pt-Y-1** preferentially binds to and is internalized by β_6_-expressing cells, indicating successful targeting. Antiproliferative effect of **oxali-Pt-Y-1** was detected when cell viability studies were carried out over 14 days. This bioconjugate approach can be adapted by incorporating alternative tumor-targeting peptides, cytotoxic agents, and labels to enhance therapeutic specificity. Higher drug loading or modified attachment sites may further optimize targeting and cytotoxicity.

Gandioso and colleagues reported a bioconjugate (**3**) composed of a photoactivatable Pt(IV) FM190 pro-drug (trans,trans,trans-[Pt(N_3_)_2_(OH)_2_(py)_2_]) in complex with the integrin-targeting c(RGDfK) peptide [126]. **3** is able to release cytotoxic Pt(II) species upon visible light irradiation. As demonstrated by photoactivation studies, upon visible light irradiation, the conjugate undergoes efficient reduction and forms Pt(II) adducts with a model nucleobase, supporting retention of desirable photochemical behavior after peptide attachment. Comparing melanoma (SK-MEL-28, high α_V_β_3_ expression) and prostate carcinoma (DU-145, low α_V_β_3_ expression) cell lines, the bioconjugate exhibited enhanced cellular uptake and preferential photocytotoxicity in highly expressing integrin cells relative to controls, consistent with integrin-mediated internalization. These outcomes indicate that the combination of an RGD peptide with a photoactivatable Pt(IV) scaffold confers a dual mode of selectivity (receptor targeting and light-controlled activation), which may achieve more localized and effective anticancer action while mitigating off-target toxicity.

The aim of Shi and colleagues’ work was the development of another targeted photoactivatable Pt(IV) prodrug by conjugating a visible-light-responsive diazido Pt(IV) complex to a cyclic peptide selective for α_6_ integrin [127]. The photoactive FM190-NHS Pt(IV) complex (trans,trans,trans-[Pt(N_3_)_2_(py)_2_(OH)(succinate-NHS)]) was covalently linked via an axial ligand to the disulphide cyclic nonapeptide c(CRWYDENAC), yielding the conjugate **Pt-cP**. Photocytotoxicity was evaluated in several human cancer cell lines (A2780, A549 and PC3), alongside healthy cells (MRC5), and Pt cellular accumulation was quantified before and after irradiation. **Pt-cP** was stable in the dark but underwent photoreduction upon blue-light irradiation, releasing Pt(II) species capable of binding guanine. The conjugate showed low dark toxicity but significantly enhanced photocytotoxicity compared to the parent complex FM190, with IC_50_ values in the low μM range and the highest potency observed in lung cancer cells (A549). **Pt-cP** exhibited lower cellular accumulation than FM190 in the dark, whereas light irradiation caused a dramatic increase in intracellular Pt levels, likely ascribable to a lower propensity of Pt(II) photoproducts to efflux from the cells than the Pt(IV) prodrug. These findings highlight the potential of photoactivable bioconjugates as an alternative strategy for achieving spatially controlled and selective anticancer activity.

Gaviglio and coworkers investigated whether conjugation of somatostatin or neurotensin analogs to a Pt(IV) prodrug enhances the antiproliferative activity in cancer cells [128]. Two peptides were employed: a pseudo-neurotensin fragment (pNT) and the somatostatin analogue octreotate (tate). Four Pt(IV)-peptide bioconjugates were synthesized by coupling one (**2**, **4**) or two peptide units (**3**, **5**) of pNT (KKPYIL) and tate (f*cyclo*(CFwKTC)T), respectively, in the axial positions. Antiproliferative activity was evaluated in several human cancer cell lines (MCF-7, Panc1, HepG2, PT45) expressing the corresponding receptors. The Pt(IV) precursor exhibited very low cytotoxicity across all tested cell lines. In contrast, all bioconjugates showed significantly enhanced antiproliferative activity, with potency gains of up to one order of magnitude. For neurotensin conjugates, the presence of one versus two peptides slightly influenced cytotoxicity, whereas octreotate conjugates showed comparable activity regardless of peptide number.

In their study, Massaguer and colleagues described the development of bioconjugates containing a picoplatin derivative and an RGD peptide [129]. Monomeric c(RGDfK) and tetrameric RAFT-{c(RGDfK)}_4_ peptides were synthesized and covalently linked to a Pt(IV) complex, resulting in **5** and **6** bioconjugates, respectively. Melanoma cells (SK-MEL-28), as an integrin-overexpressing model, and pancreatic cancer cells (CAPAN-1) and fibroblasts (1BR3G), as negative controls, were selected. Cellular uptake and internalization were investigated. Cytotoxic experiments showed that antitumor activity of picoplatin in melanoma cells increased by 2.6-fold in **5** and by 20-fold when in **6**. The results demonstrated that **6** markedly enhanced Pt accumulation and antiproliferative efficacy in SK-MEL-28, whereas minimal activity was observed in control cells. Overall, this work highlights the advantages of multivalent RGD-based targeting strategies.

When appropriately engineered, Pt(IV)-peptide conjugates can enhance cellular uptake and confer functional selectivity (RTPs and CPPs), inducing immune activation, while retaining the prodrug character of the Pt(IV) scaffold. Crucial consideration must be given to redox stability of the Pt(IV) center, choice of the peptide ligand, nature of the linker and axial functionalization, all critical parameters that determine biological outcome. Insufficient control over the reduction kinetics of bioconjugates can lead to premature Pt(IV) activation, undermining targeting strategies, and consequently improving nonspecific effects. Collectively, reported results demonstrate that conjugation of peptides to Pt(IV) pro-drugs represents a highly design-dependent strategy to improve the therapeutic index of Pt-based chemotherapy.

## 4. Gold-Peptide Conjugates

### 4.1. Au(I)-Peptide Conjugates

Gold(I) complexes emerged as a distinctive class of metal-based anticancer agents whose mechanism of action differs substantially from that of classical platinum drugs. Rather than targeting DNA, most Au(I) compounds exert their cytotoxic effects through high-affinity interactions with soft biological nucleophiles, in particular thiol- and selenol-containing proteins [68,69]. Among the biological targets identified so far, thioredoxin reductase (TrxR) is considered one of the most relevant [133]. This selenoenzyme plays a central role in maintaining intracellular redox homeostasis by regulating the thioredoxin system and protecting cells from oxidative damage. Coordination of Au(I) species to the highly nucleophilic selenocysteine residue located in the active site of TrxR results in enzyme inhibition, disruption of redox balance, and accumulation of reactive oxygen species (ROS) [63]. In cancer cells, elevated ROS levels can induce oxidative damage to proteins, lipids, and nucleic acids, ultimately triggering mitochondrial dysfunction and apoptotic cell death [134]. In addition to TrxR, Au(I) complexes may interact with other thiol-rich proteins involved in antioxidant defense, protein folding, and mitochondrial metabolism, further contributing to oxidative stress and cellular dysfunction. Among the sulfur-rich proteins with which gold complexes can interact, serum albumin has also been identified [68]. Consequently, inhibition of thiol- and selenol-dependent pathways represents a distinctive and promising strategy for selective anticancer intervention.

**Figure 6:**
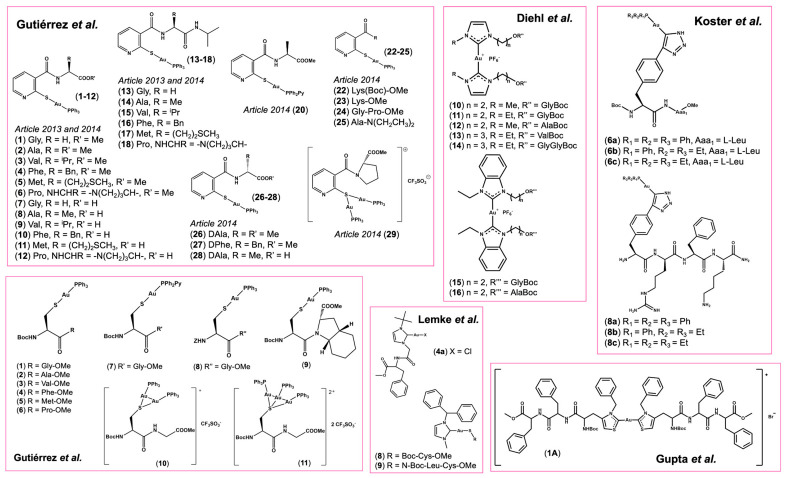
Structure of all discussed Au(I)-peptide bioconjugates [135,136,137,138,139,140,141].

Within this framework, amino acid/peptide conjugation was explored as a strategy to improve the delivery, stability, and selectivity of Au(I) complexes, while preserving their unique biological targets. Studies present in the literature are reported below (Table 3 and Figure 6).

Gutiérrez et al. reported the synthesis of a new family of gold(I) thiolate complexes containing amino acid moieties obtained by functionalization of the nicotinic acid thiolate precursor [Au(SpyCOOH)(PPh_3_)] [135]. Coupling reactions with amino acid methyl esters afforded the amino acid ester derivatives **1**–**6** (Gly, Ala, Val, Phe, Met, and Pro analogues), formulated as [Au{SpyCONHCH(R)COOMe}(PPh_3_)]. Subsequent hydrolysis generated the corresponding water-soluble amino acid derivatives **7**–**12**, while further coupling with isopropylamine yielded the amide analogues **13**–**18**. Structural characterization confirmed the integrity of the Au-S and Au-P bonds and demonstrated retention of the stereochemical configuration of the amino acids during the synthetic procedures. Crystallographic studies on complexes **1** and **3** revealed the expected linear coordination geometry around Au(I).

In a follow-up study, Gutierrez et al. evaluated the antiproliferative activity of previously reported complexes (**1**–**18**) and expanded the library with new derivatives [136]. Previous compounds were complemented with phosphine-modified analogues (**20**), a dinuclear gold(I) complex (**21**), Lys-containing derivatives (**22**–**23**), the Gly-Pro dipeptide conjugate (**24**), a tertiary amide derivative (**25**), D-amino acid analogues (**26**–**28**), and the dinuclear Pro-containing hybrid complex (**29**). Cytotoxicity studies against different tumor cell lines (A549, Jurkat, and MiaPaca2) showed that most compounds exhibited low micromolar IC_50_ values, generally lower than CDDP. Structure-activity relationship analyses revealed that ester derivatives were consistently more active than the corresponding acids or amides, while incorporation of Pro markedly enhanced cytotoxicity. The dinuclear complexes (**29**) displayed some of the most promising activity, suggesting a beneficial effect of increasing the gold content per molecule.

Gutiérrez and coworkers also reported a series of Au(I)-peptide bioconjugates (**1**–**11**) in which the gold center is directly coordinated to Cys-containing dipeptides [137]. Starting from Cys, authors protected the amino group with Boc or Z, coupled various proteinogenic and non-proteinogenic amino acid esters (Gly, Ala, Val, Phe, Met, Pro, and a conformationally restricted octahydro-indole derivative) and introduced orthogonal modifications, such as different phosphine ligands, and variations in the number of Au atoms per molecule. All bioconjugates displayed in vitro cytotoxicity against three human tumor cell lines (A549, MiaPaCa2 and Jurkat) with IC_50_ values in low- and sub-micromolar range, and highest values for **1**–**6**. Compared with CDDP, the gold-peptide conjugates were markedly more active, especially in platinum-resistant cell lines (A549). Structure-activity relationship (SAR) analysis showed that complexes bearing Gly (**1**, **7**, **8**, **10**, **11**) or Pro (**6**) gave the best potency, and the addition of the octahydro-indole ester (**9**) further enhanced activity in MiaPaCa2.

Another approach was used by Köster and coworkers, who for the first time reported organometallic (phosphine)Au(I) compounds conjugated to MTPs by a spontaneous click chemistry approach [138]. Authors attached di- and tetra-peptides to a (phosphine)gold(I)azide ligand which exhibits antimitochondrial activity, obtaining the final di- (**6a**–**c**) and tetra-bioconjugates (**8a**–**c**). Their effects on cell growth and viability of MCF-7 and HT-29 cell lines, as well as on normal human skin fibroblasts (GM5756), were investigated, yielding values comparable to those of established clinical drugs CDDP and auranofin, with the best results obtained for bioconjugate **6c**. Cellular uptake assays in HT-29 cells demonstrated that **6c** exhibited the highest uptake compared to the other bioconjugates, which is reflected in its more potent cytotoxic effects. Furthermore, all compounds showed strong inhibitory effects on TrxR. The evaluation of their biological activity in cancer cells p53-mutant MDA-MB231 indicated a concentration-dependent decrease in mitochondrial respiration for compounds **8b**, **8c**, **6b**, **6c**, and, to a lesser extent, for **6a** and **8a** in a CDDP-resistant p53-mutant cancer cell line, highlighting their ability to overcome CDDP resistance.

An alternative synthetic strategy was subsequently proposed by Lemke and colleagues, who described the synthesis and characterization of a series of bioconjugates bearing one amino acid attached through its N-terminus to an NHC-ligand (**4a**, **4c**, **5a**, **5c**) and one amino acid or a dipeptide conjugated to an Au center via a Cys residue (**8**, **9**) [139]. Bioconjugate activity was evaluated in cancer cell lines (HeLa, HepG2, HT-29), indicating that for the amino acid and peptide conjugates **8** and **9**, only slight differences in activity could be detected compared to NHC gold(I) chloride **6a**. These compounds showed activity comparable to the CDDP. It is worth noting that amino acid transporters are over-expressed on some tumor cell lines, thus the aminoacidic moiety of **8** and **9** could improve the selectivity towards tumor cells over healthy cells.

The study of Diehl et al. reported the synthesis and characterization of new bioconjugates involving direct conjugation of amino acids/peptides to different NHC ligands [140]. These bioconjugates (**10**–**16**) represent the first bifunctional Au(I)-based compounds encompassing two amino acids/peptides.

Furthermore, the investigation of Gupta and colleagues aimed to develop a potential anticancer metallodrug by incorporating two scaffolds containing non-proteinogenic amino acid thiazolylalanine into a Phe-Phe dipeptide to enable carbene formation on the thiazolium ring [141]. The tripeptide ligand was conjugated to Au(I) center to obtain a biscarbene complex (**1A**). Biological studies elucidated that **1A** readily internalized into cells, selectively reducing the viability of breast cancer cell line (MCF-7), and no cytotoxic effect was observed for other cells (MDA-MB-231, WiDr, Colo320HSR, 22RV1). Biological assays to detect and quantify apoptosis confirm programmed cell death as bioconjugates mode of action.

In conclusion, in the reported bioconjugates, the peptide can be conjugated to Au(I) center by directly sulfur-containing amino acids or attached to an NHC-ligand. NHC ligands confer increased kinetic stability, reducing premature ligand exchange in biological media. More, sulfur-containing ligands prevent the interaction of gold with sulfur-rich proteins, exploiting the trans-effect.

### 4.2. Au(III)-Peptide Conjugates

Au(III) complexes are commonly regarded as redox-activated systems, and their anticancer activity depends on intracellular reduction processes that generate Au(I) species or other reactive intermediates capable of interacting with biological targets [133].

The biological application of Au(III) complexes is intrinsically challenged by their high redox lability under physiological conditions, which often leads to reduction by endogenous reducing agents such as glutathione, Cys, ascorbate, and other sulfur-containing biomolecules [142]. Consequently, stabilization of the Au(III) oxidation state remains one of the major challenges in the development of gold-based therapeutics. To address this issue, considerable efforts have been devoted to the design of ligand frameworks capable of increasing kinetic and thermodynamic stability while preserving the possibility of intracellular activation [142]. Cyclometalated ligands, multidentate chelators, and peptide-based coordination environments have all been explored as effective strategies to modulate the redox behavior of Au(III) complexes. Regarding peptide conjugation, the nature of the peptide and the coordination mode adopted, may contribute to stabilization of the Au(III) oxidation state, improve water solubility, enhance tumor selectivity, and promote cellular uptake. Nevertheless, the design of effective Au(III)-peptide bioconjugates requires careful control of both redox activation and targeting properties.

**Figure 7:**
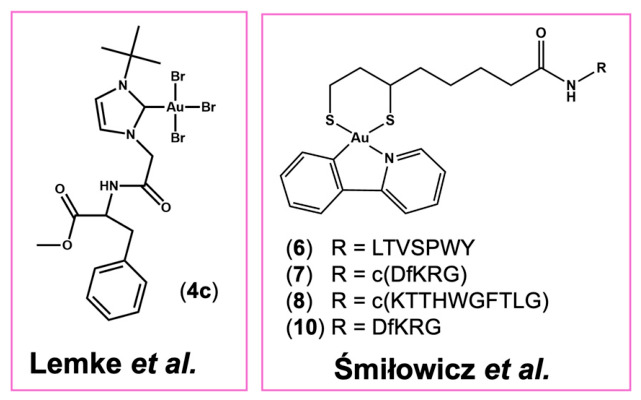
Structure of all discussed Au(III)-peptide bioconjugates [139,143].

Despite the relatively limited number of examples reported (Table 4 and Figure 7) compared to Au(I)-based systems, Au(III)-peptide conjugates represent a promising, albeit challenging, area of research in the development of gold-based anticancer agents.

In their study, Lemke and colleagues describe Au(I) (above reported) and Au(III) complexes, in which gold coordinates NHC-amino acid conjugates [139]. The Au(III) bioconjugate **4c** tested in different cancer cell lines (HeLa, HepG2, and HT-29) showed reduced activity with respect to the Au(I) counterpart and CDDP.

Śmiłowicz’s group reported a slightly different approach for the synthesis of its gold(III) conjugates [143]. Using a combination of solution and solid phase strategy, the authors attached a cyclometalated Au(III) complex (Au(ppy)Cl_2_, ppy = 2-phenyl-pyridine) via lipoic acid to linear (LTVSPWY and DfKRG) and cyclic peptides (KTTHWGFTLG and DfKRG). All bioconjugates (**6**–**8**, **10**) were tested against two human breast cancer cell lines (MCF-7 and MDA-MB-231) and a normal fibroblast line (GM5657T), displaying greater cytotoxicity than gold precursor and CDDP. Bioconjugate **7** was the most active, showing approximately 10-fold higher potency than the linear analogue **10**. This enhanced activity is attributed to peptide cyclization, which stabilizes the RGD motif and promotes integrin-mediated cell targeting, thereby increasing cellular uptake and tumor-cell selectivity. The work demonstrates that conjugating Au(III) complexes to cell-targeting peptides can substantially improve anticancer efficacy.

Overall, the limited literature indicates that the biological performance of Au(III)-peptide conjugates strongly depends on complex stability and conjugation strategy. While Au(III) complexes may be prone to redox instability, as highlighted by Lemke et al., the manuscript from Śmiłowicz’s group demonstrates that cyclometalation and peptide targeting can effectively stabilize Au(III) and enhance anticancer activity and selectivity. These findings suggest that, despite their underexplored nature, Au(III)-peptide bioconjugates remain promising candidates for targeted cancer therapy when supported by appropriate ligand and peptide design.

## 5. Discussion

A critical evaluation of bioconjugates discussed in this review reveals that peptide conjugation generally exerts a beneficial effect on the biological properties of metal complexes. Nevertheless, the nature of the peptide strongly influences the outcome. CPPs frequently enhance cellular internalization and intracellular metal accumulation, particularly in platinum systems. However, this increased uptake is often accompanied by limited tumor selectivity because CPP-mediated transport is not restricted to cancer cells. In contrast, RTPs may provide greater selectivity by exploiting the overexpression of specific receptors on tumor cells, although their effect on total cellular uptake may be less pronounced. These observations indicate that cellular internalization and biological selectivity should not be regarded as equivalent parameters and that optimization of one does not necessarily improve the other.

The impact of peptide conjugation on the cellular uptake mechanisms of metal complexes should be taken into account. Classical Pt(II) drugs, such as CDDP and carboplatin, are relatively small molecules and may exploit both passive diffusion and carrier-mediated transport pathways, including copper transport systems [18]. The introduction of bulky and often highly polar peptide moieties can alter these transport properties, potentially reducing access to uptake mechanisms available to the parent complexes. Consequently, peptide conjugation is expected to be most beneficial when the peptide actively promotes receptor-mediated recognition and internalization.

An additional aspect that deserves consideration is the potential role of peptide conjugation in overcoming resistance to metal-based chemotherapy. Resistance to platinum drugs may arise through multiple mechanisms [144,145]. In principle, peptide conjugation could help circumvent some of these mechanisms by promoting alternative uptake routes, increasing intracellular accumulation, or redirecting the metal complex toward different subcellular compartments [146,147]. Indeed, some amino acid-, CPP- and RTP-containing conjugates have shown promising activity in resistant cancer models [102,137,138]. However, the currently available evidence remains relatively limited, and only a small number of studies directly investigated resistance mechanisms in detail, even if peptide-mediated delivery represents a promising strategy for mitigating multidrug resistance.

It should also be recognized that the remarkable clinical success of CDDP is not only related to its anticancer efficacy but also to its relatively simple synthesis, straightforward manufacturing, and low production cost. In this context, the development of more complex metal-peptide bioconjugates should not necessarily be viewed as an attempt to replace CDDP solely on the basis of potency. Rather, these systems aim to address major limitations of conventional platinum chemotherapy, including systemic toxicity, poor selectivity, and drug resistance. Consequently, the added synthetic complexity and production costs of peptide-conjugated systems may be justified if they provide meaningful improvements in therapeutic index and patient outcome.

This review reveals that the biological evidence supporting metal-peptide bioconjugates is highly heterogeneous. Not all investigations employ the same level of biological validation. While DNA interaction studies remain useful for understanding the chemical behavior of platinum complexes, DNA damage alone cannot be considered a reliable predictor of antitumor efficacy. Therefore, the most informative studies are those that progress beyond mechanistic investigations and evaluate biological activity in increasingly complex models. In this respect, the relatively small number of platinum bioconjugate studies that include in vivo validation deserve particular attention [103,109,118,119,120,121]. By contrast, none of the reviewed Au(I)- or Au(III)-peptide conjugates have yet progressed to animal studies. This observation suggests that pharmacokinetic factors, biodistribution, and systemic toxicity remain largely unexplored for gold-peptide bioconjugates.

From authors’ perspective, some studies stand out not only because of their biological results, but also because of their conceptual novelty. Particularly intriguing examples include the self-assembling short aliphatic peptide systems [109], the branched Y-shaped peptide scaffold [125] and the photoactivatable Pt(IV) systems [126,127].

The more represented RTP sequences in both platinum and gold bioconjugates display RGD peptide motifs useful for increasing cellular uptake and activity in specific models, although relevant evidence for receptor-mediated selectivity is still to be improved [105,106,107,108,116,126,129,143].

One more question emerging from the reviewed literature is whether peptide conjugation improves the biological activity of the precursor metal complex. The answer appears to be positive in several cases, although far from universal. Among Pt-based systems, improved activity relative to the corresponding precursor was reported in several studies (Table 5) [101,102,103,105,106,108,109,115,117,118,119,120,121,122,129]. Au-based systems display a more complex behavior. In two cases [136,143], their biological activity exceeded that of CDDP, but not necessarily that of the corresponding gold precursor. Collectively, these findings indicate that peptide conjugation does not systematically enhance cytotoxic potency and that the biological contribution of the peptide must be evaluated on a case-by-case basis.

Finally, a consideration that clearly emerges from Pt(IV)-based systems concerns the extent of peptide functionalization. Axial functionalization represents one of the major advantages of Pt(IV) prodrugs and provides a versatile platform for the introduction of targeting moieties. However, studies directly comparing mono-functionalized and bis-functionalized Pt(IV) conjugates suggest that increasing the number of peptide units does not necessarily improve biological performance. In three works [116,117,118,119], conjugates bearing two peptide sequences generally displayed reduced cytotoxicity compared with their mono-functionalized counterparts, whereas, in one [128], no substantial differences between the two approaches have been reported. Interestingly, in another work [129] the functionalization at only one axial position involves a marked improvement when moving from a peptide monomer to a tetrameric construct. Thus, functionalization of both axial positions may negatively affect cellular uptake, intracellular trafficking, or drug activation, ultimately reducing antitumor efficacy.

## 6. Conclusions and Future Perspectives

Studies discussed in this review collectively demonstrate that peptide conjugation represents a powerful, yet non-trivial, strategy for modulating the biological behavior of platinum- and gold-based anticancer agents. The literature examined highlights that peptides have been successfully employed to preserve the metal center features and improve the selective targeting and cellular/subcellular uptake.

However, peptide conjugation does not universally translate into enhanced anticancer activity, and its biological impact strongly depends on the specific metal complex, peptide sequence, and overall conjugate design. While several platinum-peptide conjugates have shown promising results, including in vivo efficacy, the development of gold-peptide systems remains largely limited to in vitro studies, leaving important pharmacological aspects unexplored.

From a forward-looking perspective, future research in metal-peptide conjugates should shift toward a design based on the metal mechanism of action. The rational integration of peptide structure, linker and metal center will be essential to improve delivery effects and pharmacological activity. Ultimately, the most promising advances are likely to arise from multifunctional systems that combine controlled activation, validated targeting, and well-defined mechanisms of action, rather than from peptide conjugation as an isolated design strategy. Furthermore, the approach of using bispecific systems that exploit the selectivity of two different peptides for receptors overexpressed in the same type of tumor cells also appears very promising for a future perspective [95]. Furthermore, the introduction of varied chemical modifications in peptide sequences [148], the use of peptidomimetics and peptoids in these systems could also represent a step forward [149,150], considering their high protease-resistance. Based on the current state of the art, several studies have reported the development of bioconjugates involving gold(III) complexes and peptidomimetics [151,152,153,154,155,156,157,158,159]; conversely no published results are available for platinum bioconjugate counterparts. Peptoids may be considered an intriguing class of peptidomimetics, and, to the best of current knowledge, the only studies reported in literature on peptoid-metal bioconjugates are limited to diagnostic purposes [160,161,162,163,164].

Continuing to improve metal-peptide conjugates is essential to finding therapies targeted to specific types of cancer, exploiting highly selective peptides for receptors overexpressed on these cells, and contextually reducing side effects [165,166].

## Figures and Tables

**Table 1:** Pt(II)-peptide bioconjugates. Note: Compound numbering has been retained as reported in the original publications. This choice was made to facilitate direct comparison with the source articles and to enable readers interested in specific studies to more easily identify and retrieve the corresponding compounds in the original literature.

Author	Pt(II)-Peptide Bioconjugate	Peptide	Pt Precursor	Cell Lines	Ref.
Robillard et al. (2000)	**1**	AEG-Gly-Arg *	Pt(en)Cl_2_ **	Not performed	[97]
Robillard et al. (2003)	**3**	AEG-Gly-Gly *	Pt(en)Cl_2_ **	DNA specificity in pUC19, HeLa	[98]
	**4**	AEG-Gly-Phe *			
	**5**	AEG-Gly-Lys *			
	**6**	AEG-Gly-Arg *			
	**7**	AEG-Gly-Ser *			
	**8**	AEG-Gly-Glu *			
Kumbhakonam et al. (2018)	**7a**	Boc-Ala	Pt(en)Cl_2_ **	SiHa	[99]
	**7b**	Boc-Leu-Leu			
	**7c**	Boc-Leu			
	**7d**	Boc-Orn			
	**7e**	Boc-Lys			
	**7f**	Orn			
	**7g**	Lys			
	**7h**	Boc-Orn(Boc)-Orn(Boc)			
	**7i**	Orn-Orn			
	**7j**	Boc-Orn(Boc)-Orn(Boc)-Orn(Boc)			
	**7k**	Orn-Orn-Orn			
	**21**	Boc-Ile-Asp			
	**26**	Boc-Asp-Leu-Phe			
	**32a**	Boc-Asp-Orn(Boc)	[Pt(en)Cl_2_]_2_ **		
	**32b**	Asp-Orn			
Mügge et al. (2023)	**28**	YGGFL	Pt(II) with an (O,S) chelating moiety	Not performed	[100]
	**29**				
	**30**				
	**32**				
Aroui et al. (2015)	**Pt-1-DMCa**	D-MCa (GDCLPHLKLCKENKDCCSKKCKRRGTNIEKRCR)	[MBL-III-7]PtCl_2_	SVGp12, U87	[101]
Wlodarczyk et al. (2018)	**Pt-NLS hybrid**	NLS (PKKKRKV)	Carboplatin	A2780, CP70, TOV-21G, SKOV3, ES-2, OV-90	[102]
Calderon et al. (2016)	**Pt-Mal-LHRH**	LHRH (pGlu-His-Trp-Ser-Tyr-DLys-Leu-Arg-Pro-Gly)	Carboplatin	4T1, MDA-MB-231, 3T3	[103]
Teles et al. (2020)	**1**	HAIYPHRH	PtCl(BPG) ***	U251, MCF-7, ADR/RES, 786-0, H460, PC-3, OVCAR-3, HaCat	[104]
Ndinguri et al. (2009)	**7**	Cyclic CNGRC	Carboplatin	PC-3	[105]
	**8**				
Chatzisideri et al. (2017)	**4**	c(RGDyK)	cyclometalated [N,C,N-Pt(II)] complex	PC3, SCOV-3, A549, MDA-MB-231, MCF- 7, U87M	[106]
Zamora et al. (2018)	**Pt-c(RGDfK)**	c(RGDfK)	PtCl(dmba)(dmso) ****	SK-MEL-28, MDA-MB-231, CAPAN-1, HUVEC	[107]
Medrano et al. (2017)	**Pt(II)-cRGD**	c(RGDfK)	trans-[PtCl_2_(NH_2_CH(CH_3_)_2_) (4-picolinic acid)]	MDA-MB-231, MCF7, A2780, SKOV3, A549	[108]
Reithofer et al. (2014)	**2**	LIVAGK-NH_2_	Oxaliplatin	HeLa, SW480, 4T1	[109]
	**3**	LIVAGD-OH			
	**4**	IVD-OH			
	**5**	IVK-NH_2_			

* AEG = N-2-aminoethyl-glycine; ** en = ethylenediamine; *** BPG = bis(2-pyridylmethyl)glycine; **** dmba = C,N-dimethylbenzylamine.

**Table 2:** Pt(IV)-peptide bioconjugate. Note: Compound numbering has been retained as reported in the original publications. This choice was made to facilitate direct comparison with the source articles and to enable readers interested in specific studies to more easily identify and retrieve the corresponding compounds in the original literature.

Author	Pt(IV)-Peptide Bioconjugate	Peptide	Pt Precursor	Cell Lines	Ref.
Medrano et al. (2017)	**Pt(IV)-cRGD**	cRGDfK	Trans-[PtCl_2_(NH_2_CH(CH_3_)_2_)(4-picolinic acid)]	Not performed	[108]
Śmiłowicz et al. (2018)	**4a**	P1 ((R)_9_-GAL)	Carboplatin	MCF-7, HepG2 and GM5657T	[115]
	**4b**	P2 ((R)_9_-GALGLP(E)_9_)			
Mukhopadhyay et al. (2008)	**2a**	RGD	Cisplatin	BCE, HMVEC, HUVEC, U87, ASPC1, MES-SA, HeLa	[116]
	**2b**	RGD			
	**3a**	NGR			
	**3b**	NGR			
	**4a**	AGR			
	**4b**	AGR			
	**5a**	Gly			
	**5b**	Gly			
	**6**	(CRGDC)c			
	**7a**	(RGDfK)c			
	**7b**	(RGDfK)c			
Abramkin et al. (2011)	**2**	TAT (YGRKKRRQRRR)	Oxaliplatin	CH1, A549, SW480, SKOV-3	[117]
	**3**				
Li et al. (2025)	**Pt(IV)-TAT**	TAT (CCYRGRKKRRQRRR)	Cisplatin	4T1, A549	[118]
Li et al. (2025)	**s-CisPt(IV)-TAT**	TAT (CCYRGRKKRRQRRR)	Cisplatin	A549, U-87 MG, PC-3	[119]
	**d-CisPt(IV)-TAT**				
Linares et al. (2023)	**C-POC**	CPP2 (DSLKSYYLQKFSWR)	Oxaliplatin	LoVo, HT29, SW620, PDOs	[120]
Jimenez-Macias et al. (2022)	**Pt(IV)-M13**	M13 (AGYLLGKINLKA*cyclo*(CAALAKKC)L)	Cisplatin	G30-LRP, G34-pCDH, GBM-X6, BT286, G9-pCDH	[121]
Graf et al. (2012)	**1-CTX**	CTX (MCMPCFTTDHQMAR)	Cisplatin	HeLa, MCF-7, A549	[122]
Wong et al. (2014)	**3a**	ANXA1 (2-12)	Cisplatin	U-87MG, MDA-MB-231, MCF-7	[123]
	**3b**	ANXA1 (2-26)			
	**3c**	WKYMVm			
	**3d**	fMLFK			
	**4**	WKYMVm			
Mayr et al. (2017)	**3**	LARLLT	Cisplatin	A431, RUMH, HCC827, H520	[124]
	**4a**	LARLLT	Oxaliplatin		
	**4b**	LARLLT			
	**5**	RTALLL			
Conibear et al. (2017)	**cis-Pt-Y-1**	P1 (RGDLATLRQL)	Cisplatin	SW480 ITGB6 low and high expression level, A431	[125]
	**oxali-Pt-Y-1**		Oxaliplatin		
Gandioso et al. (2015)	**3**	c(RGDfK)	FM190 *	SK-MEL-28, DU-145, MDA-MB-468	[126]
Shi et al. (2019)	**Pt-cP**	c(CRWYDENAC)	FM190-NHS **	A2780, A549, PC3, MRC5	[127]
Gaviglio et al. (2012)	**2**	pNT (KKPYIL)	Cisplatin	MCF-7, Panc1, HepG2, PT45	[128]
	**3**				
	**4**	tate (f*cyclo*(CFwKTC)T)			
	**5**				
Massaguer et al. (2015)	**5**	c(RGDfK)	Picoplatin	SK-MEL-28, CAPAN-1, 1BR3G	[129]
	**6**	RAFT-{c(RGDfK)}_4_			

* FM190 = (trans,trans,trans-[Pt(N_3_)_2_(OH)_2_(py)_2_]); ** FM190-NHS = (trans,trans,trans-[Pt(N_3_)_2_(py)_2_(OH)(succinate-NHS)]).

**Table 3:** Au(I)-peptide bioconjugates. Note: Compound numbering has been retained as reported in the original publications. This choice was made to facilitate direct comparison with the source articles and to enable readers interested in specific studies to more easily identify and retrieve the corresponding compounds in the original literature.

Author	Au(I)-Peptide Bioconjugate	Peptide	Au Precursor	Cell Lines	Ref.
Gutiérrez et al. (2013)	**1**	Gly-OMe	[Au(SpyCOOH)(PPh_3_)]	Not performed	[135]
	**2**	Ala-OMe			
	**3**	Val-OMe			
	**4**	Phe-OMe			
	**5**	Met-OMe			
	**6**	Pro-OMe			
	**7**	Gly			
	**8**	Ala			
	**9**	Val			
	**10**	Phe			
	**11**	Met			
	**12**	Pro			
	**13**	Gly-X *			
	**14**	Ala-X *			
	**15**	Val-X *			
	**16**	Phe-X *			
	**17**	Met-X *			
	**18**	Pro-X *			
Gutiérrez et al. (2014)	**1**	Gly-OMe	[Au(SpyCOOH)(PPh_3_)]	A549, MiaPaca2, Jukart, 293T, R69	[136]
	**2**	Ala-OMe			
	**3**	Val-OMe			
	**4**	Phe-OMe			
	**5**	Met-OMe			
	**6**	Pro-OMe			
	**7**	Gly			
	**8**	Ala			
	**9**	Val			
	**10**	Phe			
	**11**	Met			
	**12**	Pro			
	**13**	Gly-X *			
	**14**	Ala-X *			
	**15**	Val-X *			
	**16**	Phe-X *			
	**17**	Met-X *			
	**18**	Pro-X *			
	**20**	Ala-OMe	[Au(SpyCOOH)(PPh_2_Py)]		
	**22**	Lys(Boc)-OMe	[Au(SpyCOOH)(PPh_3_)]		
	**23**	Lys-OMe			
	**24**	Gly-Pro-OMe			
	**25**	Gly-N(CH_2_CH_3_)_2_			
	**26**	DAla-OMe			
	**27**	DPhe-OMe			
	**28**	DAla			
	**29**	Pro-OMe	[Au_2_(SpyCOOH)(PPh_3_)_2_]		
Gutiérrez et al. (2015)	**1**	Boc-Cys-Gly-OMe	AuCl(PPh_3_)	A549, MiaPaCa2, Jurkat	[137]
	**2**	Boc-Cys-Ala-OMe			
	**3**	Boc-Cys-Val-OMe			
	**4**	Boc-Cys-Phe-OMe			
	**5**	Boc-Cys-Met-OMe			
	**6**	Boc-Cys-Pro-OMe			
	**7**	Boc-Cys-Gly-OMe	AuCl(PPh_2_Py)		
	**8**	Z-Cys-Gly-OMe **	AuCl(PPh_3_)		
	**9**	Boc-Cys-X-OMe ***			
	**10**	Boc-Cys-Gly-OMe	[AuCl(PPh_3_)]_2_		
	**11**	Boc-Cys-Gly-OMe	[AuCl(PPh_3_)]_3_		
Köster et al. (2012)	**6a**	Phe-Leu	(phosphine)gold(I)azide	MCF-7, HT-29, GM5756, p53-mutant MDA-MB231	[138]
	**6b**				
	**6c**				
	**8a**	Phe-D-Arg-Phe-Lys			
	**8b**				
	**8c**				
Lemke et al. (2009)	**4a**	Phe-OMe	NHC gold (imidazole-derived)	HeLa, HepG2, HT-29	[139]
	**8**	Boc-Cys-OMe			
	**9**	Boc-Leu-Cys-OMe			
Diehl et al. (2017)	**10**	Gly-Boc	NHC gold (imidazole-derivative)	Not performed	[140]
	**11**	Gly-Boc			
	**12**	Ala-Boc			
	**13**	Val-Boc			
	**14**	Gly-Gly-Boc			
	**15**	Gly-Boc	NHC gold (benzimidazol-derivative)		
	**16**	Ala-Boc			
Gupta et al. (2019)	**1A**	Boc-Z-Phe-Phe-OMe ****	[AuCl(tht)] *****	MCF-7, MDA-MB-231, WiDr, Colo320HSR, 22RV1	[141]

* X = isopropylamine; ** Z = benzyloxycarbonyl; *** X = octahydro-indole derivative; **** Z = thiazolylalanine; ***** tht = tetrahydrothiophene.

**Table 4:** Au(III)-peptide bioconjugates. Note: Compound numbering has been retained as reported in the original publications. This choice was made to facilitate direct comparison with the source articles and to enable readers interested in specific studies to more easily identify and retrieve the corresponding compounds in the original literature.

Author	Au(III)-Peptide Bioconjugate	Peptide	Au Precursor	Cell Lines	Ref.
Lemke et al. (2009)	**4c**	Phe-OMe	NHC gold (imidazole-derived)	HeLa, HepG2, HT-29	[139]
Śmiłowicz et al. (2019)	**6**	LTVSPWY	Au(ppy)Cl_2_ *	MCF-7, MDA-MB-231 and GM5657T	[143]
	**7**	c(DfKRG)			
	**8**	c(KTTHWGFTLG)			
	**10**	DfKRG			

* ppy = 2-phenyl-pyridine.

**Table 5:** Summarizing table.

Metal	Author	Peptide	Bioconjugate vs. Precursor: Improvement	Refs.
Pt(II)	Robillard et al.	Di-peptide	✕	[97,98]
	Kumbhakonam et al.	Amino acid or Di-/Tri-peptide	✕	[99]
	Mügge et al.	RTP	NP	[100]
	Aroui et al.	CPP	✓	[101]
	Wlodarczyk et al.	CPP	✓	[102]
	Calderon et al.	RTP	✓	[103]
	Teles et al.	RTP	✕	[104]
	Ndinguri et al.	RTP	✓	[105]
	Chatzisideri et al.	RTP	✓	[106]
	Zamora et al.	RTP	✕	[107]
	Medrano et al.	RTP	✓	[108]
	Reithofer et al.	Self-assembling aliphatic peptides	✓	[109]
Pt(IV)	Medrano et al.	RTP	NP	[108]
	Śmiłowicz et al.	CPP	✓	[115]
	Mukhopadhyay et al.	RTP	✕	[116]
	Abramkin et al.	CPP	✓	[117]
	Li et al.	CPP	✓	[118,119]
	Linares et al.	CPP	=	[120]
	Jimenez-Macias et al.	CPP	✓	[121]
	Graf et al.	RTP	✓	[122]
	Wong et al.	RTP	✕	[123]
	Mayr et al.	RTP	✕	[124]
	Conibear et al.	RTP	✕	[125]
	Gandioso et al.	RTP	✕	[126]
	Shi et al.	RTP	✕	[127]
	Gaviglio et al.	RTP	✕	[128]
	Massaguer et al.	RTP	✓	[129]
Au(I)	Gutiérrez et al.	Amino acid	✓	[135,136]
	Gutiérrez et al.	Di-peptide	✓	[137]
	Köster et al.	Di-/Tetra-peptide	=	[138]
	Lemke et al.	Di-peptide	=	[139]
	Diehl et al.	Di-peptide	=	[140]
	Gupta et al.	Di-peptide	✕	[141]
Au(III)	Lemke et al.	Amino acid	✕	[139]
	Śmiłowicz et al.	RTP	✓	[143]

In this table: RTP = receptor-targeting peptide; CPP = cell-penetrating peptide; ✓ represents an improvement in the cytotoxicity of the bioconjugate compared to the precursor complex; ✕ represents a worsening in the cytotoxicity of the bioconjugate compared to the precursor complex; = represents an almost comparable cytotoxicity of the bioconjugate compared to the precursor complex; NP = not performed.

## Data Availability

No new data were created or analyzed in this study. Data sharing does not apply to this article.

## Abbreviations

The following abbreviations are used in this manuscript:

AEG	N-2-aminoethyl-glycine
APN	Aminopeptidase N
BBB	Blood–brain barrier
Boc	Tert-butyloxycarbonyl
BPG	Bis(2-pyridylmethyl)glycine
BRLs	Bispecific Radioligands
CDDP	Cisplatin
CPP	Cell-Penetrating Peptide
dmba	C,N-dimethylbenzylamine
EGFR	Epidermal Growth Factor Receptor
en	Ethylenediamine
FT-IR	Fourier Transform Infrared Spectroscopy
ICP-MS	Inductively Coupled Plasma Mass Spectrometry
LHRH	Luteinizing Hormone-Releasing Hormone
MRI	Magnetic Resonance Imaging
MTP	Mitochondria-Targeting Peptide
NHC	N-Heterocyclic Carbene
NLS	Nuclear Localization Signal
NMR	Nuclear Magnetic Resonance
PBMC	Peripheral Blood Mononuclear Cell
PDO	Patients-Derived Organoid
PEG	Polyethylene glycol
pNT	Pseudo-Neurotensin
ppy	2-phenyl-pyridine
RP	Radiopharmaceutical
RTP	Receptor-Targeting Peptide
SAR	Structure–Activity Relationship
SPPS	Solid-Phase Peptide Synthesis
TfrR	Transferrin Receptor
TrxR	Thioredoxin Reductase
TME	Tumor Microenvironment

## References

[1] Lippert B. (2013). Uses of Metal Compounds in Medicine. Reference Module in Chemistry, Molecular Sciences and Chemical Engineering.

[2] Drakesmith H., Prentice A.M. (2012). Hepcidin and the Iron-Infection Axis. Science.

[3] Gamberi T., Hanif M. (2022). Metal-Based Complexes in Cancer Treatment. Biomedicines.

[4] Kim H.-K., Lee G.H., Chang Y. (2018). Gadolinium as an MRI Contrast Agent. Future Med. Chem..

[5] Hheidari A., Mohammadi J., Ghodousi M., Mahmoodi M., Ebrahimi S., Pishbin E., Rahdar A. (2024). Metal-Based Nanoparticle in Cancer Treatment: Lessons Learned and Challenges. Front. Bioeng. Biotechnol..

[6] Dong L., Ding J., Zhu L., Liu Y., Gao X., Zhou W. (2023). Copper Carbonate Nanoparticles as an Effective Biomineralized Carrier to Load Macromolecular Drugs for Multimodal Therapy. Chin. Chem. Lett..

[7] Nie Y., Li D., Peng Y., Wang S., Hu S., Liu M., Ding J., Zhou W. (2020). Metal Organic Framework Coated MnO2 Nanosheets Delivering Doxorubicin and Self-Activated DNAzyme for Chemo-Gene Combinatorial Treatment of Cancer. Int. J. Pharm..

[8] Rosenberg B., Vancamp L., Trosko J.E., Mansour V.H. (1969). Platinum Compounds: A New Class of Potent Antitumour Agents. Nature.

[9] Lippert B. (1999). Cisplatin: Chemistry and Biochemistry of a leading Anticancer Drug.

[10] Kelland L. (2007). The Resurgence of Platinum-Based Cancer Chemotherapy. Nat. Rev. Cancer.

[11] Yao X., Panichpisal K., Kurtzman N., Nugent K. (2007). Cisplatin Nephrotoxicity: A Review. Am. J. Med. Sci..

[12] Knox R.J., Friedlos F., Lydall D.A., Roberts J.J. (1986). Mechanism of Cytotoxicity of Anticancer Platinum Drugs: Evidence That Cis-Diamminedichloroplatinum(II) and Cis-Diammine-(1,1-Cyclobutanedicarboxylato)Platinum(II) Differ Only in the Kinetics of Their Interaction with DNA. Cancer Res..

[13] Lim K.-H., Huang M.-J., Lin H.-C., Su Y.-W., Chang Y.-F., Lin J., Chang M.-C., Hsieh R.-K. (2004). Hypersensitivity Reactions to Oxaliplatin: A Case Report and the Success of a Continuous Infusional Desensitization Schedule. Anti-Cancer Drugs.

[14] Calvert H. (2019). The Clinical Development of Carboplatin—A Personal Perspective. Inorg. Chim. Acta.

[15] Grothey A., Goldberg R.M. (2004). A Review of Oxaliplatin and Its Clinical Use in Colorectal Cancer. Expert Opin. Pharmacother..

[16] Jung Y., Lippard S.J. (2007). Direct Cellular Responses to Platinum-Induced DNA Damage. Chem. Rev..

[17] Fuertes M.A., Alonso C., Pérez J.M. (2003). Biochemical Modulation of Cisplatin Mechanisms of Action:  Enhancement of Antitumor Activity and Circumvention of Drug Resistance. Chem. Rev..

[18] Arnesano F., Nardella M.I., Natile G. (2018). Platinum Drugs, Copper Transporters and Copper Chelators. Coord. Chem. Rev..

[19] Shruthi S., Bhasker Shenoy K. (2024). Cisplatin Resistance in Cancer Therapy: Causes and Overcoming Strategies. ChemistrySelect.

[20] Shahlaei M., Asl S.M., Derakhshani A., Kurek L., Karges J., Macgregor R., Saeidifar M., Kostova I., Saboury A.A. (2024). Platinum-Based Drugs in Cancer Treatment: Expanding Horizons and Overcoming Resistance. J. Mol. Struct..

[21] Cucciolito M.E., D’Amora A., De Feo G., Ferraro G., Giorgio A., Petruk G., Monti D.M., Merlino A., Ruffo F. (2018). Five-Coordinate Platinum(II) Compounds Containing Sugar Ligands: Synthesis, Characterization, Cytotoxic Activity, and Interaction with Biological Macromolecules. Inorg. Chem..

[22] Xu Z., Wang Z., Deng Z., Zhu G. (2021). Recent Advances in the Synthesis, Stability, and Activation of Platinum(IV) Anticancer Prodrugs. Coord. Chem. Rev..

[23] Wexselblatt E., Gibson D. (2012). What Do We Know about the Reduction of Pt(IV) pro-Drugs?. J. Inorg. Biochem..

[24] Iova V., Tincu R.C., Scrobota I., Tudosie M.S. (2025). Pt(IV) Complexes as Anticancer Drugs and Their Relationship with Oxidative Stress. Biomedicines.

[25] Gibson D. (2019). Multi-Action Pt(IV) Anticancer Agents; Do We Understand How They Work?. J. Inorg. Biochem..

[26] Giandomenico C.M., Abrams M.J., Murrer B.A., Vollano J.F., Rheinheimer M.I., Wyer S.B., Bossard G.E., Higgins J.D. (1995). Carboxylation of Kinetically Inert Platinum(IV) Hydroxy Complexes. An Entr.acte.ee into Orally Active Platinum(IV) Antitumor Agents. Inorg. Chem..

[27] Hambley T.W., Battle A.R., Deacon G.B., Lawrenz E.T., Fallon G.D., Gatehouse B.M., Webster L.K., Rainone S. (1999). Modifying the Properties of Platinum(IV) Complexes in Order to Increase Biological Effectiveness. J. Inorg. Biochem..

[28] Bhargava A., Vaishampayan U.N. (2009). Satraplatin: Leading the New Generation of Oral Platinum Agents. Expert Opin. Investig. Drugs.

[29] Liang W., Huang Y., Wang Y., Lu D., Sun Q. (2025). Research Progress of Platinum-Based Complexes in Lung Cancer Treatment: Mechanisms, Applications, and Challenges. Int. J. Mol. Sci..

[30] Kee J.X., Yau J.N.N., Kumar Muthuramalingam R.P., Wang X., Chng W.H., Lopez-Sanchez A., Tay K.K.W., Deng L.-W., Gibson D., Bertrand H.C. (2025). Colorectal Cancer at the Crossroads: The Good, the Bad, and the Future of Platinum-Based Drugs. Chem. Rev..

[31] Yusoh N.A., Ahmad H., Vallis K.A., Gill M.R. (2025). Advances in Platinum-Based Cancer Therapy: Overcoming Platinum Resistance through Rational Combinatorial Strategies. Med. Oncol..

[32] Shahsavani M.B., Heidari M., Yousefi R., Moosavi-Movahedi A.A. (2025). Platinum-Based Chemotherapeutics in the Modern Era: From Classical DNA-Targeting Mechanisms to Next-Generation Innovations in Cancer Therapy. Chem. Biol. Drug Des..

[33] Khoury A., Deo K.M., Aldrich-Wright J.R. (2020). Recent Advances in Platinum-Based Chemotherapeutics That Exhibit Inhibitory and Targeted Mechanisms of Action. J. Inorg. Biochem..

[34] Lee S.Y., Kim C.Y., Nam T.-G. (2020). Ruthenium Complexes as Anticancer Agents: A Brief History and Perspectives. Drug Des. Dev. Ther..

[35] Scattolin T., Voloshkin V.A., Visentin F., Nolan S.P. (2021). A Critical Review of Palladium Organometallic Anticancer Agents. Cell Rep. Phys. Sci..

[36] Unavane S., Patil R., Syed S., Jain H.K. (2025). Exploring the Therapeutic Potential of Copper and Cobalt Complexes as Anticancer Agents: A Comprehensive Review. Transit Met. Chem..

[37] Santini C., Pellei M., Gandin V., Porchia M., Tisato F., Marzano C. (2014). Advances in Copper Complexes as Anticancer Agents. Chem. Rev..

[38] Nobili S., Mini E., Landini I., Gabbiani C., Casini A., Messori L. (2010). Gold Compounds as Anticancer Agents: Chemistry, Cellular Pharmacology, and Preclinical Studies. Med. Res. Rev..

[39] Paprocka R., Wiese-Szadkowska M., Janciauskiene S., Kosmalski T., Kulik M., Helmin-Basa A. (2022). Latest Developments in Metal Complexes as Anticancer Agents. Coord. Chem. Rev..

[40] Kean W.F., Kean I.R.L. (2008). Clinical Pharmacology of Gold. Inflammopharmacology.

[41] Pricker S.P. (1996). Medical Uses of Gold Compounds: Past, Present and Future. Gold. Bull..

[42] Shaw C.F. (1999). Gold-Based Therapeutic Agents. Chem. Rev..

[43] Bernhard G.C. (1982). Auranofin Therapy in Rheumatoid Arthritis. J. Lab. Clin. Med..

[44] Ott I. (2009). On the Medicinal Chemistry of Gold Complexes as Anticancer Drugs. Coord. Chem. Rev..

[45] Gabbiani C., Casini A., Messori L. (2007). Gold(III) Compounds as Anticancer Drugs. Gold. Bull..

[46] Wani M.Y., Malik M.A. (2021). Gold and Its Complexes in Anticancer Chemotherapy.

[47] Lu Y., Ma X., Chang X., Liang Z., Lv L., Shan M., Lu Q., Wen Z., Gust R., Liu W. (2022). Recent Development of Gold (I) and Gold (III) Complexes as Therapeutic Agents for Cancer Diseases. Chem. Soc. Rev..

[48] Moreno-Alcántar G., Picchetti P., Casini A. (2023). Gold Complexes in Anticancer Therapy: From New Design Principles to Particle-Based Delivery Systems. Angew. Chem. Int. Ed..

[49] Kumar P., Navya P.N., Begum A., Ojha R., Plebanski M., Bhargava S.K. (2025). Unveiling the Anticancer Potential of Gold-Sulfur Complexes: Mechanisms, Delivery Strategies, and Clinical Progression. Coord. Chem. Rev..

[50] Marinova P., Blazheva D., Nikolova S. (2026). Anticancer Applications of Gold Complexes: Structure–Activity Review. Appl. Sci..

[51] Zhang M., Yi X., Xie S., Zhan Q. (2026). A Critical Review of Gold(I) Complexes as Anticancer Agents: From Canonical Mechanisms to Immune Activation. Bioorganic Chem..

[52] Ferraro G., Giorgio A., Mansour A.M., Merlino A. (2019). Protein-Mediated Disproportionation of Au(i): Insights from the Structures of Adducts of Au(Iii) Compounds Bearing N,N-Pyridylbenzimidazole Derivatives with Lysozyme. Dalton Trans..

[53] Shaw C.F. (1989). The Protein Chemistry of Antiarthritic Gold(I) Thiolates and Related Complexes. Comments Inorg. Chem..

[54] Messori L., Abbate F., Marcon G., Orioli P., Fontani M., Mini E., Mazzei T., Carotti S., O’Connell T., Zanello P. (2000). Gold(III) Complexes as Potential Antitumor Agents: Solution Chemistry and Cytotoxic Properties of Some Selected Gold(III) Compounds. J. Med. Chem..

[55] Ronconi L., Giovagnini L., Marzano C., Bettìo F., Graziani R., Pilloni G., Fregona D. (2005). Gold Dithiocarbamate Derivatives as Potential Antineoplastic Agents:  Design, Spectroscopic Properties, and in Vitro Antitumor Activity. Inorg. Chem..

[56] Wang Y., He Q.-Y., Sun R.W.-Y., Che C.-M., Chiu J.-F. (2005). Gold(III) Porphyrin 1a Induced Apoptosis by Mitochondrial Death Pathways Related to Reactive Oxygen Species. Cancer Res..

[57] Che C.-M., Wai-Yin Sun R., Yu W.-Y., Ko C.-B., Zhu N., Sun H. (2003). Gold(III) Porphyrins as a New Class of Anticancer Drugs: Cytotoxicity, DNA Binding and Induction of Apoptosis in Human Cervix Epitheloid Cancer Cells. Chem. Commun..

[58] Marcon G., Carotti S., Coronnello M., Messori L., Mini E., Orioli P., Mazzei T., Cinellu M.A., Minghetti G. (2002). Gold(III) Complexes with Bipyridyl Ligands:  Solution Chemistry, Cytotoxicity, and DNA Binding Properties. J. Med. Chem..

[59] Messori L., Marcon G., Cinellu M.A., Coronnello M., Mini E., Gabbiani C., Orioli P. (2004). Solution Chemistry and Cytotoxic Properties of Novel Organogold(III) Compounds. Bioorganic Med. Chem..

[60] Coronnello M., Mini E., Caciagli B., Cinellu M.A., Bindoli A., Gabbiani C., Messori L. (2005). Mechanisms of Cytotoxicity of Selected Organogold(III) Compounds. J. Med. Chem..

[61] Gabbiani C., Guerri A., Cinellu M.A., Messori L. (2010). Dinuclear Gold(III) Complexes as Potential Anticancer Agents: Structure, Reactivity and Biological Profile of a Series of Gold(III) Oxo-Bridged Derivatives. Open Crystallogr. J..

[62] Casini A., Hartinger C., Gabbiani C., Mini E., Dyson P.J., Keppler B.K., Messori L. (2008). Gold(III) Compounds as Anticancer Agents: Relevance of Gold–Protein Interactions for Their Mechanism of Action. J. Inorg. Biochem..

[63] Bindoli A., Rigobello M.P., Scutari G., Gabbiani C., Casini A., Messori L. (2009). Thioredoxin Reductase: A Target for Gold Compounds Acting as Potential Anticancer Drugs. Coord. Chem. Rev..

[64] Nardon C., Boscutti G., Gabbiani C., Massai L., Pettenuzzo N., Fassina A., Messori L., Fregona D. (2017). Cell and Cell-Free Mechanistic Studies on Two Gold(III) Complexes with Proven Antitumor Properties. Eur. J. Inorg. Chem..

[65] Marcon G., Messori L., Orioli P., Cinellu M.A., Minghetti G. (2003). Reactions of Gold(III) Complexes with Serum Albumin. Eur. J. Biochem..

[66] Yei Ho S., Tiekink E.R.T. (2005). 79Au Gold-Based Metallotherapeutics: Use and Potential. Metallotherapeutic Drugs and Metal-Based Diagnostic Agents.

[67] Pratesi A., Gabbiani C., Michelucci E., Ginanneschi M., Papini A.M., Rubbiani R., Ott I., Messori L. (2014). Insights on the Mechanism of Thioredoxin Reductase Inhibition by Gold N-Heterocyclic Carbene Compounds Using the Synthetic Linear Selenocysteine Containing C-Terminal Peptide hTrxR(488-499): An ESI-MS Investigation. J. Inorg. Biochem..

[68] Pratesi A., Cirri D., Fregona D., Ferraro G., Giorgio A., Merlino A., Messori L. (2019). Structural Characterization of a Gold/Serum Albumin Complex. Inorg. Chem..

[69] Giorgio A., Merlino A. (2020). Gold Metalation of Proteins: Structural Studies. Coord. Chem. Rev..

[70] Wang L., Wang N., Zhang W., Cheng X., Yan Z., Shao G., Wang X., Wang R., Fu C. (2022). Therapeutic Peptides: Current Applications and Future Directions. Signal Transduct. Target Ther..

[71] Duffuler P., Bhullar K.S., de Campos Zani S.C., Wu J. (2022). Bioactive Peptides: From Basic Research to Clinical Trials and Commercialization. J. Agric. Food Chem..

[72] Berillo D., Yeskendir A., Zharkinbekov Z., Raziyeva K., Saparov A. (2021). Peptide-Based Drug Delivery Systems. Medicina.

[73] Tesauro D., Accardo A., Diaferia C., Milano V., Guillon J., Ronga L., Rossi F. (2019). Peptide-Based Drug-Delivery Systems in Biotechnological Applications: Recent Advances and Perspectives. Molecules.

[74] Capasso D., de Paola I., Liguoro A., Gatto A.D., Gaetano S.D., Guarnieri D., Saviano M., Zaccaro L. (2014). RGDechi-hCit: Avβ3 Selective Pro-Apoptotic Peptide as Potential Carrier for Drug Delivery into Melanoma Metastatic Cells. PLoS ONE.

[75] Banks W.A. (2015). Peptides and the Blood–Brain Barrier. Peptides.

[76] Stalmans S., Bracke N., Wynendaele E., Gevaert B., Peremans K., Burvenich C., Polis I., Spiegeleer B.D. (2015). Cell-Penetrating Peptides Selectively Cross the Blood-Brain Barrier In Vivo. PLoS ONE.

[77] Dai J., Ashrafizadeh M., Aref A.R., Sethi G., Ertas Y.N. (2024). Peptide-Functionalized, -Assembled and -Loaded Nanoparticles in Cancer Therapy. Drug Discov. Today.

[78] Sharma R., Borah S.J., Bhawna, Kumar S., Gupta A., Singh P., Goel V.K., Kumar R., Kumar V. (2022). Functionalized Peptide-Based Nanoparticles for Targeted Cancer Nanotherapeutics: A State-of-the-Art Review. ACS Omega.

[79] Omidian H., Wilson R.L., Castejon A.M. (2025). Recent Advances in Peptide-Loaded PLGA Nanocarriers for Drug Delivery and Regenerative Medicine. Pharmaceuticals.

[80] Del Gatto A., Di Pietro P., Saviano M., Tomasello M.F., Pappalardo G., Snyders R., Forte G., Satriano C., Zaccaro L. (2025). Bioinspired RGD-Functionalized Gold Nanoparticles for Integrin-Driven Interaction with Melanoma Cells. Int. J. Nanomed..

[81] Depalo N., Corricelli M., De Paola I., Valente G., Iacobazzi R.M., Altamura E., Debellis D., Comegna D., Fanizza E., Denora N. (2017). NIR Emitting Nanoprobes Based on Cyclic RGD Motif Conjugated PbS Quantum Dots for Integrin-Targeted Optical Bioimaging. ACS Appl. Mater. Interfaces.

[82] Valente G., Depalo N., de Paola I., Iacobazzi R.M., Denora N., Laquintana V., Comparelli R., Altamura E., Latronico T., Altomare M. (2016). Integrin-Targeting with Peptide-Bioconjugated Semiconductor-Magnetic Nanocrystalline Heterostructures. Nano Res..

[83] Di Pietro P., Zaccaro L., Comegna D., Del Gatto A., Saviano M., Snyders R., Cossement D., Satriano C., Rizzarelli E. (2016). Silver Nanoparticles Functionalized with a Fluorescent Cyclic RGD Peptide: A Versatile Integrin Targeting Platform for Cells and Bacteria. RSC Adv..

[84] Aronson M.R., Medina S.H., Mitchell M.J. (2021). Peptide Functionalized Liposomes for Receptor Targeted Cancer Therapy. APL Bioeng..

[85] Sonju J.J., Dahal A., Singh S.S., Jois S.D. (2021). Peptide-Functionalized Liposomes as Therapeutic and Diagnostic Tools for Cancer Treatment. J. Control. Release.

[86] Heppeler A., Froidevaux S., Eberle A.N., Maecke H.R. (2000). Receptor Targeting for Tumor Localisation and Therapy with Radiopeptides. Curr. Med. Chem..

[87] Aina O.H., Sroka T.C., Chen M.-L., Lam K.S. (2002). Therapeutic Cancer Targeting Peptides. Pept. Sci..

[88] Zhang H., Zhang Y., Zhang C., Yu H., Ma Y., Li Z., Shi N. (2023). Recent Advances of Cell-Penetrating Peptides and Their Application as Vectors for Delivery of Peptide and Protein-Based Cargo Molecules. Pharmaceutics.

[89] Cerrato C.P., Langel Ü. (2022). An Update on Cell-Penetrating Peptides with Intracellular Organelle Targeting. Expert Opin. Drug Deliv..

[90] Goldfarb D.S., Gariépy J., Schoolnik G., Kornberg R.D. (1986). Synthetic Peptides as Nuclear Localization Signals. Nature.

[91] Kim S., Nam H.Y., Lee J., Seo J. (2020). Mitochondrion-Targeting Peptides and Peptidomimetics: Recent Progress and Design Principles. Biochemistry.

[92] Yan Y.-Q., Wang H., Zhao Y. (2022). Radiolabeled Peptide Probe for Tumor Imaging. Chin. Chem. Lett..

[93] Bolzati C., Salvarese N., Carpanese D., Seraglia R., Meléndez-Alafort L., Rosato A., Capasso D., Saviano M., Del Gatto A., Comegna D. (2018). [99mTc][Tc(N)PNP43]-Labeled RGD Peptides As New Probes for a Selective Detection of Avβ3 Integrin: Synthesis, Structure–Activity and Pharmacokinetic Studies. J. Med. Chem..

[94] Wang X., Li C., Wang Y., Chen H., Zhang X., Luo C., Zhou W., Li L., Teng L., Yu H. (2022). Smart Drug Delivery Systems for Precise Cancer Therapy. Acta Pharm. Sin. B.

[95] Bentivoglio V., D’Ippolito E., Nayak P., Giorgio A., Lauri C. (2025). Bispecific Radioligands (BRLs): Two Is Better Than One. J. Clin. Med..

[96] Zuccolo M., Arrighetti N., Perego P., Colombo D. (2022). Recent Progresses in Conjugation with Bioactive Ligands to Improve the Anticancer Activity of Platinum Compounds. Curr. Med. Chem..

[97] Robillard M.S., Valentijn A.R.P.M., Meeuwenoord N.J., van der Marel G.A., van Boom J.H., Reedijk J. (2000). The First Solid-Phase Synthesis of a Peptide-Tethered Platinum(II) Complex. Angew. Chem. Int. Ed..

[98] Robillard M.S., Davies N.P., van der Marel G.A., van Boom J.H., Reedijk J., Murray V. (2003). The Interaction of Peptide-Tethered Platinum(II) Complexes with DNA. J. Inorg. Biochem..

[99] Kumbhakonam S., Vellaisamy K., Saroj S., Venkatesan N., Karunagaran D., Kannoth Manheri M. (2018). Serine- and Threonine-Derived Diamine Equivalents for Site-Specific Incorporation of Platinum Centers in Peptides, and the Anticancer Potential of These Conjugates. New J. Chem..

[100] Mügge C., Nowak N., Strack M., Metzler-Nolte N., Weigand W. (2023). Synthetic Approaches towards Peptide-Conjugates of Pt(II) Compounds with an (*O*,*S*) Chelating Moiety. Eur. J. Inorg. Chem..

[101] Aroui S., Dardevet L., Ajmia W.B., De Boisvilliers M., Perrin F., Laajimi A., Boumendjel A., Kenani A., Muller J.M., De Waard M. (2015). A Novel Platinum–Maurocalcine Conjugate Induces Apoptosis of Human Glioblastoma Cells by Acting through the ROS-ERK/AKT-P53 Pathway. Mol. Pharm..

[102] Wlodarczyk M.T., Dragulska S.A., Camacho-Vanegas O., Dottino P.R., Jarzęcki A.A., Martignetti J.A., Mieszawska A.J. (2018). Platinum(II) Complex-Nuclear Localization Sequence Peptide Hybrid for Overcoming Platinum Resistance in Cancer Therapy. ACS Biomater. Sci. Eng..

[103] Calderon L.E., Keeling J.K., Rollins J., Black C.A., Collins K., Arnold N., Vance D.E., Ndinguri M.W. (2017). Pt-Mal-LHRH, a Newly Synthesized Compound Attenuating Breast Cancer Tumor Growth and Metastasis by Targeting Overexpression of the LHRH Receptor. Bioconj. Chem..

[104] Teles C.M., Antunes V.U., Cardoso R.S., Candido T.Z., Lima C.S.P., Ruiz A.L.T.G., Juliano M.A., Favaro D.C., Abbehausen C. (2020). Functionalization of New Anticancer Pt(II) Complex with Transferrin Receptor Binding Peptide. Inorg. Chim. Acta.

[105] Ndinguri M.W., Solipuram R., Gambrell R.P., Aggarwal S., Hammer R.P. (2009). Peptide Targeting of Platinum Anti-Cancer Drugs. Bioconj. Chem..

[106] Chatzisideri T., Thysiadis S., Katsamakas S., Dalezis P., Sigala I., Lazarides T., Nikolakaki E., Trafalis D., Gederaas O.A., Lindgren M. (2017). Synthesis and Biological Evaluation of a Platinum(II)-c(RGDyK) Conjugate for Integrin-Targeted Photodynamic Therapy. Eur. J. Med. Chem..

[107] Zamora A., Gandioso A., Massaguer A., Buenestado S., Calvis C., Hernández J.L., Mitjans F., Rodríguez V., Ruiz J., Marchán V. (2018). Toward Angiogenesis Inhibitors Based on the Conjugation of Organometallic Platinum(II) Complexes to RGD Peptides. ChemMedChem.

[108] Medrano M.A., Morais M., Ferreira V.F.C., Correia J.D.G., Paulo A., Santos I., Navarro-Ranninger C., Valdes A.A., Casini A., Mendes F. (2017). Nonconventional Trans-Platinum Complexes Functionalized with RDG Peptides: Chemical and Cytototoxicity Studies. Eur. J. Inorg. Chem..

[109] Reithofer M.R., Chan K.-H., Lakshmanan A., Lam D.H., Mishra A., Gopalan B., Joshi M., Wang S., Hauser C.A. (2014). Ligation of Anti-Cancer Drugs to Self-Assembling Ultrashort Peptides by Click Chemistry for Localized Therapy. Chem. Sci..

[110] Robillard M.S., Bacac M., van den Elst H., Flamigni A., van der Marel G.A., van Boom J.H., Reedijk J. (2003). Automated Parallel Solid-Phase Synthesis and Anticancer Screening of a Library of Peptide-Tethered Platinum(II) Complexes. J. Comb. Chem..

[111] Gibson D. (2016). Platinum(IV) Anticancer Prodrugs—Hypotheses and Facts. Dalton Trans..

[112] Navas F., Chocarro-Calvo A., Iglesias-Hernández P., Fernández-García P., Morales V., García-Martínez J.M., Sanz R., De La Vieja A., García-Jiménez C., García-Muñoz R.A. (2024). Promising Anticancer Prodrugs Based on Pt(IV) Complexes with Bis-Organosilane Ligands in Axial Positions. J. Med. Chem..

[113] Chen S., Zhou Q., Ng K.-Y., Xu Z., Xu W., Zhu G. (2024). Advances in Technical Strategies for Monitoring the Reduction of Platinum(Iv) Complexes. Inorg. Chem. Front..

[114] Li X., Liu Y., Tian H. (2018). Current Developments in Pt(IV) Prodrugs Conjugated with Bioactive Ligands. Bioinorg. Chem. Appl..

[115] Śmiłowicz D., Metzler-Nolte N. (2018). Synthesis of Monofunctional Platinum(IV) Carboxylate Precursors for Use in Pt(IV)–Peptide Bioconjugates. Dalton Trans..

[116] Mukhopadhyay S., Barnés C.M., Haskel A., Short S.M., Barnes K.R., Lippard S.J. (2008). Conjugated Platinum(IV)−Peptide Complexes for Targeting Angiogenic Tumor Vasculature. Bioconj. Chem..

[117] Abramkin S., Valiahdi S.M., Jakupec M.A., Galanski M.S., Metzler-Nolte N., Keppler B.K. (2012). Solid-Phase Synthesis of Oxaliplatin–TAT Peptide Bioconjugates. Dalton Trans..

[118] Li R., Chong C., Liu M., Yin X., Li Y., Li Y., Yao Q., Mu Y., Zhang C. (2025). Platinum(IV) Prodrug-Coupled TAT Nuclear-Targeting Peptide for Drug Delivery and High Antitumor Efficacy with Low Toxicity. ACS Appl. Mater. Interfaces.

[119] Li R., Yin X., Chong C., Li Y., Yao Q., Mu Y., Zhang C. (2025). Exploring the Relationships between Targeted Structures and Antitumor Activities of Nuclear-Targeted Polypeptide-Functionalized Platinum(IV) Prodrugs. Mol. Pharm..

[120] Linares J., Varese M., Sallent-Aragay A., Méndez A., Palomo-Ponce S., Iglesias M., Batlle E., Pisonero J., Montagut C., Giralt E. (2023). Peptide–Platinum(IV) Conjugation Minimizes the Negative Impact of Current Anticancer Chemotherapy on Nonmalignant Cells. J. Med. Chem..

[121] Jimenez-Macias J.L., Lee Y.-C., Miller E., Finkelberg T., Zdioruk M., Berger G., Farquhar C.E., Nowicki M.O., Cho C.-F., Fedeles B.I. (2022). A Pt(IV)-Conjugated Brain Penetrant Macrocyclic Peptide Shows Pre-Clinical Efficacy in Glioblastoma. J. Control. Release.

[122] Graf N., Mokhtari T.E., Papayannopoulos I.A., Lippard S.J. (2012). Platinum(IV)-Chlorotoxin (CTX) Conjugates for Targeting Cancer Cells. J. Inorg. Biochem..

[123] Wong D.Y.Q., Wong D.Y.Q. (2018). Immuno-Chemotherapeutic Platinum(IV) Prodrugs of Cisplatin as Multimodal Anticancer Agents. Rethinking Platinum Anticancer Drug Design: Towards Targeted and Immuno-Chemotherapeutic Approaches.

[124] Mayr J., Hager S., Koblmüller B., Klose M.H.M., Holste K., Fischer B., Pelivan K., Berger W., Heffeter P., Kowol C.R. (2017). EGFR-Targeting Peptide-Coupled Platinum(IV) Complexes. J. Biol. Inorg. Chem..

[125] Conibear A.C., Hager S., Mayr J., Klose M.H.M., Keppler B.K., Kowol C.R., Heffeter P., Becker C.F.W. (2017). Multifunctional Avβ6 Integrin-Specific Peptide–Pt(IV) Conjugates for Cancer Cell Targeting. Bioconj. Chem..

[126] Gandioso A., Shaili E., Massaguer A., Artigas G., González-Cantó A., Woods J.A., Sadler P.J., Marchán V. (2015). An Integrin-Targeted Photoactivatable Pt(IV) Complex as a Selective Anticancer pro-Drug: Synthesis and Photoactivation Studies. Chem. Commun..

[127] Shi H., Wang Q., Venkatesh V., Feng G., Young S., Romero-Canelón L., Zeng I., Sadler J. (2019). Photoactive Platinum(IV) Complex Conjugated to a Cancer-Cell-Targeting Cyclic Peptide. Dalton Trans..

[128] Gaviglio L., Gross A., Metzler-Nolte N., Ravera M. (2012). Synthesis and in Vitro Cytotoxicity of Cis,Cis,Trans-Diamminedichloridodisuccinatoplatinum(Iv)–Peptidebioconjugates. Metallomics.

[129] Massaguer A., González-Cantó A., Escribano E., Barrabés S., Artigas G., Moreno V., Marchán V. (2015). Integrin-Targeted Delivery into Cancer Cells of a Pt (IV) pro-Drug through Conjugation to RGD-Containing Peptides. Dalton Trans..

[130] Mikuła-Pietrasik J., Witucka A., Pakuła M., Uruski P., Begier-Krasińska B., Niklas A., Tykarski A., Książek K. (2019). Comprehensive Review on How Platinum- and Taxane-Based Chemotherapy of Ovarian Cancer Affects Biology of Normal Cells. Cell. Mol. Life Sci..

[131] Lotti F., Jarrar A.M., Pai R.K., Hitomi M., Lathia J., Mace A., Gantt G.A., Sukhdeo K., DeVecchio J., Vasanji A. (2013). Chemotherapy Activates Cancer-Associated Fibroblasts to Maintain Colorectal Cancer-Initiating Cells by IL-17A. J. Exp. Med..

[132] Che Y., Wang J., Li Y., Lu Z., Huang J., Sun S., Mao S., Lei Y., Zang R., Sun N. (2018). Cisplatin-Activated PAI-1 Secretion in the Cancer-Associated Fibroblasts with Paracrine Effects Promoting Esophageal Squamous Cell Carcinoma Progression and Causing Chemoresistance. Cell Death Dis..

[133] Bertrand B., Casini A. (2014). A Golden Future in Medicinal Inorganic Chemistry: The Promise of Anticancer Gold Organometallic Compounds. Dalton Trans..

[134] Jia J.-J., Geng W.-S., Wang Z.-Q., Chen L., Zeng X.-S. (2019). The Role of Thioredoxin System in Cancer: Strategy for Cancer Therapy. Cancer Chemother. Pharmacol..

[135] Gutiérrez A., Bernal J., Villacampa M.D., Cativiela C., Laguna A., Gimeno M.C. (2013). Synthesis of New Gold(I) Thiolates Containing Amino Acid Moieties with Potential Biological Interest. Inorg. Chem..

[136] Gutiérrez A., Gracia-Fleta L., Marzo I., Cativiela C., Laguna A., Gimeno M.C. (2014). Gold(I) Thiolates Containing Amino Acid Moieties. Cytotoxicity and Structure–Activity Relationship Studies. Dalton Trans..

[137] Gutiérrez A., Marzo I., Cativiela C., Laguna A., Gimeno M.C. (2015). Highly Cytotoxic Bioconjugated Gold(I) Complexes with Cysteine-Containing Dipeptides. Chem. A Eur. J..

[138] Köster S.D., Alborzinia H., Can S., Kitanovic I., Wölfl S., Rubbiani R., Ott I., Riesterer P., Prokop A., Merz K. (2012). A Spontaneous Gold (I)-Azide Alkyne Cycloaddition Reaction Yields Gold-Peptide Bioconjugates Which Overcome Cisplatin Resistance in a P53-Mutant Cancer Cell Line. Chem. Sci..

[139] Lemke J., Pinto A., Niehoff P., Vasylyeva V., Metzler-Nolte N. (2009). Synthesis, Structural Characterisation and Anti-Proliferative Activity of NHC Gold Amino Acid and Peptide Conjugates. Dalton Trans..

[140] Diehl T., Krause M.T., Ueberlein S., Becker S., Trommer A., Schnakenburg G., Engeser M. (2017). Synthesis of Hydroxyl-Functionalized N-Heterocyclic Carbene Gold (i) Complexes and Peptide Conjugates. Dalton Trans..

[141] Gupta A., Nigam S., Avasthi I., Sharma B., Ateeq B., Verma S. (2019). Caspase-3 Mediated Programmed Cell Death by a Gold-Stabilised Peptide Carbene. Bioorganic Med. Chem. Lett..

[142] Radisavljević S., Petrović B. (2020). Gold(III) Complexes: An Overview on Their Kinetics, Interactions With DNA/BSA, Cytotoxic Activity, and Computational Calculations. Front. Chem..

[143] Śmiłowicz D., Slootweg J.C., Metzler-Nolte N. (2019). Bioconjugation of Cyclometalated Gold(III) Lipoic Acid Fragments to Linear and Cyclic Breast Cancer Targeting Peptides. Mol. Pharm..

[144] Ohmichi M., Hayakawa J., Tasaka K., Kurachi H., Murata Y. (2005). Mechanisms of Platinum Drug Resistance. Trends Pharmacol. Sci..

[145] Galluzzi L., Senovilla L., Vitale I., Michels J., Martins I., Kepp O., Castedo M., Kroemer G. (2012). Molecular Mechanisms of Cisplatin Resistance. Oncogene.

[146] Soler M., Feliu L., Planas M., Ribas X., Costas M. (2016). Peptide-Mediated Vectorization of Metal Complexes: Conjugation Strategies and Biomedical Applications. Dalton Trans..

[147] Albada B., Metzler-Nolte N. (2016). Organometallic–Peptide Bioconjugates: Synthetic Strategies and Medicinal Applications. Chem. Rev..

[148] Comegna D., Zannetti A., Del Gatto A., De Paola I., Russo L., Di Gaetano S., Liguoro A., Capasso D., Saviano M., Zaccaro L. (2017). Chemical Modification for Proteolytic Stabilization of the Selective αv β3 Integrin RGDechi Peptide: In Vitro and in Vivo Activities on Malignant Melanoma Cells. J. Med. Chem..

[149] Giorgio A., Del Gatto A., Pennacchio S., Saviano M., Zaccaro L. (2023). Peptoids: Smart and Emerging Candidates for the Diagnosis of Cancer, Neurological and Autoimmune Disorders. Int. J. Mol. Sci..

[150] Del Gatto A., Cobb S.L., Zhang J., Zaccaro L. (2021). Editorial: Peptidomimetics: Synthetic Tools for Drug Discovery and Development. Front. Chem..

[151] Nardon C., Fregona D. (2016). Gold(III) Complexes in the Oncological Preclinical Arena: From Aminoderivatives to Peptidomimetics. Curr. Top. Med. Chem..

[152] Celegato M., Fregona D., Mongiat M., Ronconi L., Borghese C., Canzonieri V., Casagrande N., Nardon C., Colombatti A., Aldinucci D. (2014). Preclinical Activity of Multiple-Target Gold(III)-Dithiocarbamato Peptidomimetics in Prostate Cancer Cells and Xenografts. Future Med. Chem..

[153] Kouodom M.N., Boscutti G., Celegato M., Crisma M., Sitran S., Aldinucci D., Formaggio F., Ronconi L., Fregona D. (2012). Rational Design of Gold(III)-Dithiocarbamato Peptidomimetics for the Targeted Anticancer Chemotherapy. J. Inorg. Biochem..

[154] Negom Kouodom M., Ronconi L., Celegato M., Nardon C., Marchiò L., Dou Q.P., Aldinucci D., Formaggio F., Fregona D. (2012). Toward the Selective Delivery of Chemotherapeutics into Tumor Cells by Targeting Peptide Transporters: Tailored Gold-Based Anticancer Peptidomimetics. J. Med. Chem..

[155] Nardon C., Schmitt S.M., Yang H., Zuo J., Fregona D., Dou Q.P. (2014). Gold(III)-Dithiocarbamato Peptidomimetics in the Forefront of the Targeted Anticancer Therapy: Preclinical Studies against Human Breast Neoplasia. PLoS ONE.

[156] Boscutti G., Nardon C., Marchiò L., Crisma M., Biondi B., Dalzoppo D., Dalla Via L., Formaggio F., Casini A., Fregona D. (2018). Anticancer Gold(III) Peptidomimetics: From Synthesis to in Vitro and Ex Vivo Biological Evaluations. ChemMedChem.

[157] Andrejević T.P., Glišić B.Đ., Djuran M.I. (2020). Amino Acids and Peptides as Versatile Ligands in the Synthesis of Antiproliferative Gold Complexes. Chemistry.

[158] Gutiérrez A., Gimeno M.C., Marzo I., Metzler-Nolte N. (2014). Synthesis, Characterization, and Cytotoxic Activity of AuI N,S-Heterocyclic Carbenes Derived from Peptides Containing L-Thiazolylalanine. Eur. J. Inorg. Chem..

[159] Gutiérrez A., Cativiela C., Laguna A., Gimeno M.C. (2016). Bioactive Gold(I) Complexes with 4-Mercaptoproline Derivatives. Dalton Trans..

[160] De León-Rodríguez L.M., Lubag A., Udugamasooriya D.G., Proneth B., Brekken R.A., Sun X., Kodadek T., Dean Sherry A. (2010). MRI Detection of VEGFR2 in Vivo Using a Low Molecular Weight Peptoid−(Gd)8-Dendron for Targeting. J. Am. Chem. Soc..

[161] Hao G., Hajibeigi A., León-Rodríguez L.M.D., Oz O.K., Sun X. (2011). Peptoid-Based PET Imaging of Vascular Endothelial Growth Factor Receptor (VEGFR) Expression. Am. J. Nucl. Med. Mol. Imaging.

[162] Maschauer S., Einsiedel J., Haubner R., Hocke C., Ocker M., Hübner H., Kuwert T., Gmeiner P., Prante O. (2010). Labeling and Glycosylation of Peptides Using Click Chemistry: A General Approach to 18F-Glycopeptides as Effective Imaging Probes for Positron Emission Tomography. Angew. Chem. Int. Ed..

[163] Maschauer S., Einsiedel J., Hocke C., Hübner H., Kuwert T., Gmeiner P., Prante O. (2010). Synthesis of a 68Ga-Labeled Peptoid−Peptide Hybrid for Imaging of Neurotensin Receptor Expression in Vivo. ACS Med. Chem. Lett..

[164] Maschauer S., Greff C., Einsiedel J., Ott J., Tripal P., Hübner H., Gmeiner P., Prante O. (2015). Improved Radiosynthesis and Preliminary in Vivo Evaluation of a 18F-Labeled Glycopeptide–Peptoid Hybrid for PET Imaging of Neurotensin Receptor 2. Bioorganic Med. Chem..

[165] Nayak P., Varani M., Giorgio A., Campagna G., Caserta D., Signore A. (2025). Luteinizing Hormone-Releasing Hormone (LHRH)-Targeted Treatment in Ovarian Cancer. Int. J. Mol. Sci..

[166] Giorgio A., Varani M., Lauri C., Bentivoglio V., Nayak P. (2025). Radiolabeled LHRH and FSH Analogues as Cancer Theranostic Agents: A Systematic Review. J. Clin. Med..

